# Peroxiredoxin-1 protects estrogen receptor α from oxidative stress-induced suppression and is a protein biomarker of favorable prognosis in breast cancer

**DOI:** 10.1186/bcr3691

**Published:** 2014-07-10

**Authors:** Patrick C O’Leary, Marta Terrile, Malgorzata Bajor, Pawel Gaj, Bryan T Hennessy, Gordon B Mills, Agnieszka Zagozdzon, Darran P O’Connor, Donal J Brennan, Kate Connor, Jane Li, Ana Maria Gonzalez-Angulo, Han-Dong Sun, Jian-Xin Pu, Fredrik Pontén, Mathias Uhlén, Karin Jirström, Dominika A Nowis, John P Crown, Radoslaw Zagozdzon, William M Gallagher

**Affiliations:** 1 Cancer Biology and Therapeutics Laboratory, UCD Conway Institute, UCD School of Biomolecular and Biomedical Science, Dublin 4, Ireland; 2 Department of Immunology, Center for Biostructure Research, Medical University of Warsaw, Banacha 1A, 02-097 Warsaw, Poland; 3 Department of Medical Oncology, Beaumont Hospital, Royal College of Surgeons in Ireland, 123 St Stephens Green, Dublin 2, Ireland; 4 Department of Systems Biology, University of Texas M. D. Anderson Cancer Center, 7455 Fannin Street, Houston, TX 77054, USA; 5 Department of Breast Medical Oncology, University of Texas M.D. Anderson Cancer Center, 1515 Holcombe Boulevard, Houston, TX 77054, USA; 6 State Key Laboratory of Phytochemistry and Plant Resources in West China, Kunming Institute of Botany, Chinese Academy of Sciences, Kunming Botanic Garden, Yunnan 650204, China; 7 Department of Genetics and Pathology, Rudbeck Laboratory, Dag Hammarskjöldsväg 20, Uppsala University, 75185 Uppsala, Sweden; 8 AlbaNova University Center, Royal Institute of Technology, Valhallavägen 79, 100 44 Stockholm, Sweden; 9 Division of Pathology, Department of Clinical Sciences, Lund University, Skåne University Hospital, Getingevägen 4, 22185 Lund, Sweden; 10 Genomic Medicine, Department of General, Transplant and Liver Surgery, Medical University of Warsaw, 1a Banacha St, F Building, 02-097 Warsaw, Poland; 11 Molecular Therapeutics for Cancer Ireland, National Institute for Cellular Biotechnology, Dublin City University, Glasnevin, Dublin 9, Ireland

## Abstract

**Introduction:**

Peroxiredoxin-1 (PRDX1) is a multifunctional protein, acting as a hydrogen peroxide (H_2_O_2_) scavenger, molecular chaperone and immune modulator. Although differential PRDX1 expression has been described in many tumors, the potential role of PRDX1 in breast cancer remains highly ambiguous. Using a comprehensive antibody-based proteomics approach, we interrogated PRDX1 protein as a putative biomarker in estrogen receptor (ER)-positive breast cancer.

**Methods:**

An anti-PRDX1 antibody was validated in breast cancer cell lines using immunoblotting, immunohistochemistry and reverse phase protein array (RPPA) technology. PRDX1 protein expression was evaluated in two independent breast cancer cohorts, represented on a screening RPPA (n = 712) and a validation tissue microarray (n = 498). *In vitro* assays were performed exploring the functional contribution of PRDX1, with oxidative stress conditions mimicked via treatment with H_2_O_2_, peroxynitrite, or adenanthin, a PRDX1/2 inhibitor.

**Results:**

In ER-positive cases, high PRDX1 protein expression is a biomarker of improved prognosis across both cohorts. In the validation cohort, high PRDX1 expression was an independent predictor of improved relapse-free survival (hazard ratio (HR) = 0.62, 95% confidence interval (CI) = 0.40 to 0.96, *P* = 0.032), breast cancer-specific survival (HR = 0.44, 95% CI = 0.24 to 0.79, *P* = 0.006) and overall survival (HR = 0.61, 95% CI = 0.44 to 0.85, *P* = 0.004). RPPA screening of cancer signaling proteins showed that ERα protein was upregulated in PRDX1 high tumors. Exogenous H_2_O_2_ treatment decreased ERα protein levels in ER-positive cells. PRDX1 knockdown further sensitized cells to H_2_O_2_- and peroxynitrite-mediated effects, whilst PRDX1 overexpression protected against this response. Inhibition of PRDX1/2 antioxidant activity with adenanthin dramatically reduced ERα levels in breast cancer cells.

**Conclusions:**

PRDX1 is shown to be an independent predictor of improved outcomes in ER-positive breast cancer. Through its antioxidant function, PRDX1 may prevent oxidative stress-mediated ERα loss, thereby potentially contributing to maintenance of an ER-positive phenotype in mammary tumors. These results for the first time imply a close connection between biological activity of PRDX1 and regulation of estrogen-mediated signaling in breast cancer.

## Introduction

Molecular classification of breast cancer cases using biomarkers in tumor cells provides an opportunity for the implementation of effective targeted treatment modalities, such as the expression of estrogen receptor (ER) and responses to endocrine therapy. However, despite the benefits gained by endocrine treatment, the long-term effectiveness of such targeted approaches is still unsatisfactory. Identifying novel biomarkers predictive of clinical outcome is desirable in order to guide clinicians in selecting new treatment options and monitoring the treatment response of patients, as well as potentially identifying new mechanisms that could lead to combinations with hormonal therapy.

Peroxiredoxins are a ubiquitous family of antioxidant enzymes, known to catalyze peroxide reduction to balance cellular hydrogen peroxide (H_2_O_2_) levels, which is essential for cell signaling and metabolism [1,2]. Of particular interest is the mammalian isoform, peroxiredoxin 1 (PRDX1), which is a multifunctional protein originally identified as an intracellular scavenger of H_2_O_2_[3]. It has been also shown to act as a molecular chaperone with the ability to modulate the actions of numerous molecules [4-8], a regulator of transcription [9], or as an immunomodulator [10]. There are multiple reports of differential PRDX1 expression in human malignancies (reviewed in [11]). However, the diversity of PRDX1 functions makes the prediction of its role in human tumors difficult. Further validation is necessary to address the importance of PRDX1 protein expression in each cancer type.

The specific role for PRDX1 in breast cancer is controversial. In an earlier study, PRDX1 protein was found to be overexpressed in malignant versus normal tissues in 21 of 24 patients, but no significant relationship was found between PRDX1 overexpression and common clinicopathological parameters of breast cancer [12]. In a cohort of 475 patients, it was reported that PRDX1 protein expression in breast cancer was not significantly associated with any clinicopathological parameter [13]. However, other studies have shown that overexpression of PRDX1 mRNA in human breast carcinoma is associated with higher tumor grade [14], and high expression of cytoplasmic PRDX1 protein correlated with increased risk of local recurrence after radiotherapy [15].

Conversely, several lines of evidence suggest that PRDX1 may act as a tumor suppressor in breast cancer. *Prdx1*-deficient mice suffer from shortened survival due to development of hemolytic anemia and multiple tumors, including mammary carcinomas [16]. PRDX1, acting as a chaperone, interacts with the c-Myc oncogene and suppresses its transcriptional activity [17]. Another proposed function for PRDX1 in breast cancer is as a sensor in H_2_O_2_-mediated stress-induced senescence [5]. Furthermore, Cao *et al.* have shown that PRDX1 protects the tumor suppressive function of PTEN phosphatase, likely due to the presence of a reactive oxygen species (ROS) sensitive cysteine in the catalytic domain, and reduces predisposition of genetically modified mice to develop *Ras*-induced mammary tumors [6]. Accordingly, a recent study suggests that high PRDX1 expression appears to be associated with less aggressive breast cancers [18].

Importantly, a number of the above biomarker studies suffer from shortcomings such as lack of appropriate antibody validation, small cohort size and/or the absence of a molecular explanation supporting the clinical data. Thus, there is an ongoing need for properly designed studies on the role of PRDX1 in breast cancer that follow the REMARK guidelines for prognostic biomarkers [19]. This is especially relevant in light of PRDX1 being considered a therapeutic target in other cancer types, as well as the recent development of adenanthin as a chemical inhibitor of PRDX1/2 [20].

Herein, we demonstrate a robust approach for interrogating the role of PRDX1 as a putative protein biomarker in breast cancer. We identify PRDX1 expression levels as an independent marker of favorable outcome in ER-positive tumors and elucidate a unique role for PRDX1 in maintaining ERα expression in breast cancer cells subjected to oxidative stress.

## Methods

### Tissue culture

ZR-75-1, T47D, MCF7 and SKBR3 cell lines were purchased from the European Collection of Cell Cultures (Wiltshire, UK). All cell lines were maintained through continuous passaging, and were confirmed to be free of contamination by *Mycoplasma spp*. Cells were maintained in DMEM (ZR-75-1 cell line) or RPMI-1640 (other cell lines) media (Sigma-Aldrich, St. Louis, MO, USA), with 10% FBS (Sigma-Aldrich), 100 U/mL penicillin, 100 μg/mL streptomycin (Gibco, Rockville, MD, USA) and 1.46 mg/mL L-glutamine (Gibco). Additionally, the growth medium for ZR-75-1 was supplemented with 1 nM β-estradiol (Sigma-Aldrich), and did not contain sodium pyruvate, which mediates elimination of H_2_O_2_ from the culture medium [21]. Tissue culture experimental techniques are further described in the Supplementary Methods (Additional file 1).

### Chemical reagents

Adenanthin has been generated as previously described [20]. H_2_O_2_ and peroxynitrite were obtained from Sigma-Aldrich and Millipore (Billerica, MA, USA), respectively.

### Antibody validation and immunohistochemistry

Antibody generation against PRDX1 was as described previously [22]. The Human Protein Atlas (HPA) Consortium carried out the initial quality control of the polyclonal anti-PRDX1 antibody (HPA007730) via optimization and testing on a variety of tissues (48 normal tissues and 16 cancer tissues) [23]. Immunoblotting and immunohistochemistry procedures are described in Supplementary Methods (Additional file 1).

### Patient cohorts

Clinical materials originating from three independent cohorts of breast cancer patients were utilized in this study. Demographic and clinical characteristics for all cohorts are presented in this study are described in Supplementary Table S1 (Additional file 2).

Cohort 1 was represented on a reverse phase protein array (RPPA) containing protein extracts from 58 breast cancer cell lines and 998 human breast tumors, with available clinicopathological data for 712 cases. The clinical samples were collected at M. D. Anderson Cancer Center (MDACC) Houston, TX, USA, Hospital Clinico Universitario de Valencia, Spain, University of British Columbia, Vancouver, BC, Canada and Baylor College of Medicine, Houston, TX, USA [24]. Complete clinical information was available for 574 patients. All tumors in this training set were collected by excision during their primary surgery, followed by freezing of the tissue. Tumor content was verified by histopathology. All tissues were collected under Institutional Review Board (IRB)-approved laboratory protocols, awarded by the M. D. Anderson Cancer Center IRB, University of British Columbia IRB, and the Hospital Clinico Universitario de Valencia IRB. Patients consented to bank their specimens. As this was a retrospective study, each IRB provided a waiver of informed consent.

Cohort 2 consisted of a tissue microarray (TMA) with 498 consecutive invasive breast cancer cases (442 in final analysis) diagnosed at the Department of Pathology, Malmö University Hospital, between 1987 and 1992 [25]. The median age at diagnosis was 65 years (range, 27 to 96) and the median follow-up time was 11 years (range, 0 to 17). Two hundred and sixty-three patients were dead at the last follow-up (December 2004), 90 of whom were considered to be as a direct result of breast cancer (breast cancer-specific death). Complete endocrine treatment data were available for 379 patients, 160 of whom received adjuvant tamoxifen. Information on adjuvant chemotherapy was available for 382 patients, of whom 23 patients received treatment. The study has been approved by the Ethics Committee at Lund University. Informed consent was obtained for all included patients and opting out was an option.

Cohort 3 was represented on a RPPA consisting of 410 primary breast tumors collected by The Cancer Genome Atlas (TCGA) Consortium. These frozen primary tumor specimens collected from newly diagnosed patients with invasive breast adenocarcinoma undergoing surgical resection and who had received no prior treatment for their disease (chemotherapy or radiotherapy). Owing to the short median overall follow-up (20 months) and the small number of overall survival events (44 out of 410), survival analysis was not carried out on this cohort. This cohort was used solely to correlate quantitative levels of PRDX1 protein expression with expression of 180+ other proteins assessed on the same array. The TCGA project collects high-quality breast tumor samples and makes available the clinical information, molecular/genomic profiling data, and histopathology slide images on the TCGA data portal [26]. The TCGA data is organized into two categories: one that is openly accessible to the public and one that has controlled access, available only to qualified researchers obligated to secure the data. The open access data set contains only information that is not individually unique and does not pose a risk of patient re-identification. All the data used within this manuscript was obtained from the open access data set and has passed the criteria for unrestricted publication with the following statement ‘No restrictions; all data available without limitations’ listed at the publication guidelines section of the TCGA data portal [26].

### Reverse phase protein array analysis

Protein was extracted from tumor tissue and cell lines and probed for protein expression by reverse phase protein array (RPPA) analysis as previously described [24,27-30]. RPPA analysis was completed independently for cohorts 1 and 3. The technique is further described in the Supplementary Methods (Additional file 1).

### Evaluation of immunohistochemical staining

Slides were scanned at 20X magnification using a ScanScope XT slide scanner (Aperio Technologies, Vista, CA, USA). The Spectrum Analysis algorithm package, ImageScope analysis software and Color Deconvolution algorithm (version 9; Aperio Technologies.) were applied to quantify immunohistochemical (IHC) staining. These algorithms were used to calculate the average positive intensity (API), as well as the area of positive staining, and the percentage of weak (1+), medium (2+), and strong (3+) positive staining. The final API was subtracted from 255, as these intensity ranges on an 8-bit scale of 0 to 255 (black to white, respectively). The maximum value from both cores for each patient was used for statistical analysis.

### Statistical analysis

All statistical analysis was carried out with PASW Statistics version 20 and R Project for Statistical Computing. Spearman’s rho test was used to compare protein expression across the recombinant cell line series in the immunoblotting and RPPA setting. In cohorts 1 and 2, the median PRDX1 expression level was used for stratification into high and low PRDX1 expression. Pearson’s χ2 test and Fisher’s exact test were used to evaluate associations between protein expression and clinicopathological variables. Kaplan-Meier, univariate and multivariate Cox regression analyses were used to illustrate differences between recurrence-free survival (RFS), breast cancer-specific survival (BCCS), and overall survival (OS) according to PRDX1 expression. In cohort 3, the Pearson’s correlation test was used to determine the correlation between PRDX1 and expression of other proteins, and the two-sided *t* test was used to identify proteins co-regulated between the lower and upper quartile of PRDX1 protein expression cases. In all experiments, a two-tailed test value of less than 0.05 was considered significant.

## Results

### Validation of PRDX1 antibody across different protein quantification platforms

**Figure 1:**
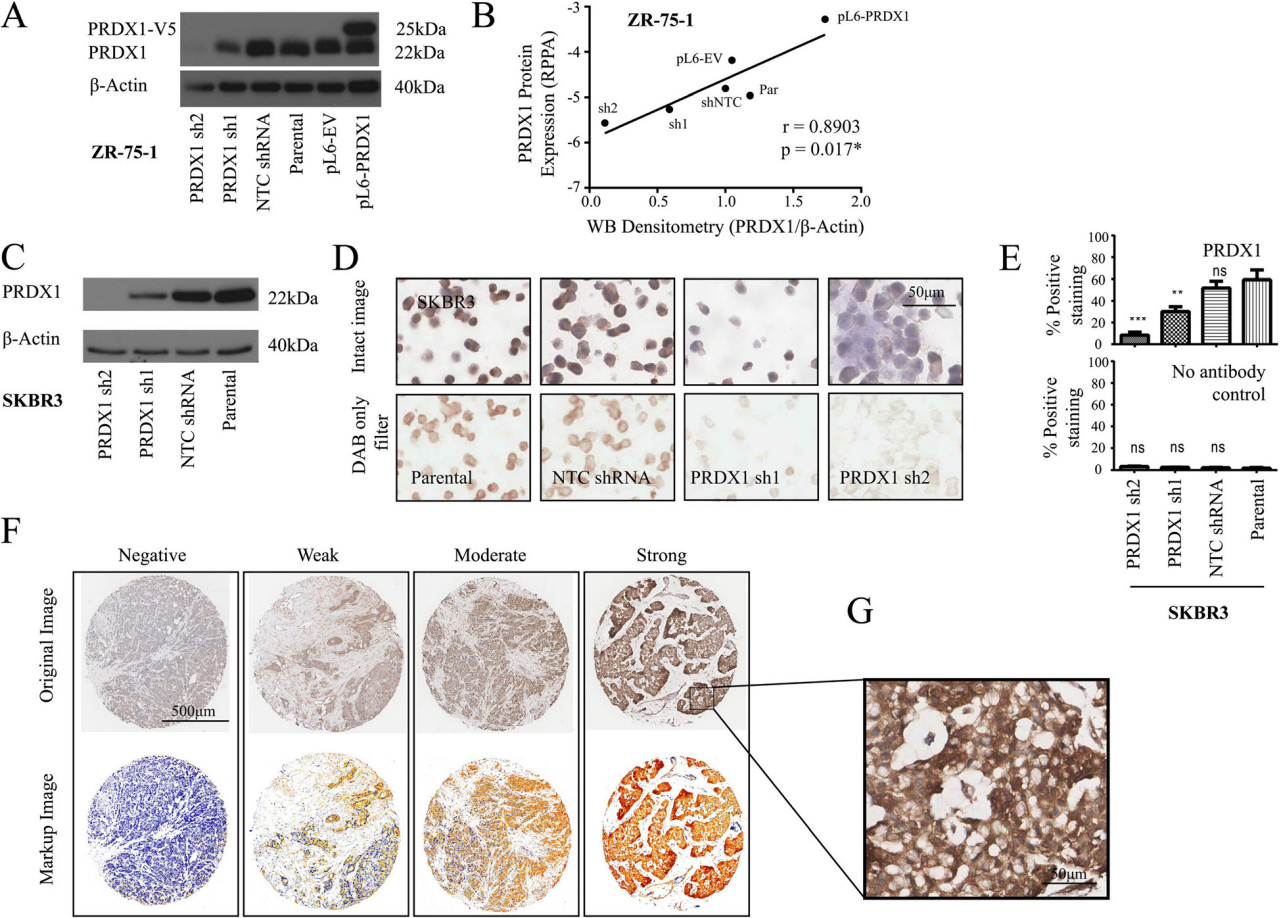
**Validation of the PRDX1 antibody specificity using immunoblotting, RPPA and IHC platforms. (A)** Immunoblotting shows a discrete signal, the intensity of which correlates with PRDX1 knockdown/overexpression across recombinant ZR-75-1 cell lines (V5-tagged PRDX1 protein runs at 25 kDa, with native protein at 22 kDa). **(B)** Correlation between Western blotting densitometry and RPPA log_2_ expression values from recombinant ZR-75-1 cell lines protein lysates. **(C)** Immunoblotting, **(D)** cell pellet arrays (upper panels: DAB and hematoxylin staining; lower panel: DAB staining only) and **(E)** automated analysis scoring from recombinant SKBR3 breast cancer cell lines (automated scoring includes data from cells stained with PRDX1 antibody or PBS-T only control). **(F)** Representation of tissue cores and associated mark-up images after automated analysis (x40 magnification). **(G)** Example of PRDX1 cytoplasmic staining. (ns: non-significant; ^**^P <0.01; ^***^P <0.001). IHC, immunohistochemistry; PBS-T, phosphate-buffered saline with 0.1% Tween-20; PRDX1, peroxiredoxin 1; RPPA, reverse phase protein arrays.

In light of conflicting results regarding the prognostic relevance of PRDX1 expression in breast cancer, a fundamental step for our study was comprehensive validation of the anti-PRDX1 antibody. Antibody specificity was confirmed in several breast cancer cell lines (T47D, ZR-75-1 and SKBR3), whereby PRDX1 expression was modified using short hairpin loop RNA (shRNA)-mediated gene knockdown and overexpression of cDNA encoding V5-tagged PRDX1, before antibody validation by immunoblotting (Figure 1A and C; complete gel displayed in Figure S1A in Additional file 3), RPPA analysis (Figure 1B) and IHC (Figure 1D and E). IHC performed on formalin-fixed paraffin-embedded (FFPE) SKBR3 cells revealed a decrease in staining intensity in cells expressing either of two shRNA molecules against PRDX1 (Figure 1D, lower panel) compared to non-targeting or parental cell controls. A significant decrease was seen in percentage 3,3-diaminobenzidine (DAB) positivity of these knockdown cells (Figure 1E). This observation confirmed the specificity of the antibody in the IHC setting prior to staining of clinical specimens. Figure 1F shows representative examples of different intensities of DAB staining, that is, expression of PRDX1 protein on TMA cores. PRDX1 protein expression was found to be predominantly cytoplasmic throughout the cores (Figure 1G). An automated algorithm was used to develop a quantitative scoring model of PRDX1 protein expression, with the respective mark-up image shown. To rule out the possibility of the antibody binding to PRDX2, a protein with high homology for PRDX1, cell lines overexpressing a pLenti6-PRDX2-V5 plasmid were generated. Modulation of PRDX1 expression levels did not affect PRDX2 protein expression, and vice versa (Figure S1D in Additional file 3). An additional PRDX1-targeting antibody was tested [18]; however, this antibody did not satisfactorily detect differential protein expression compared to mRNA expression measured in the shRNA-expressing cell lines (Figure S1B-C in Additional file 3). These extensive validation steps allow us a high level of certainty that the antibody used is specific to PRDX1 in all techniques used throughout the study.

### Identification of PRDX1 protein as a biomarker of good prognosis in ER-positive breast tumors

RPPA technology is a particularly useful tool to aid in the identification and validation of protein and phosphoprotein biomarkers using limited amounts of protein from clinical samples. PRDX1 protein expression was assessed on a RPPA cohort with clinical data available for 712 primary human breast tumors. Protein expression data was dichotomized based on the median PRDX1 expression values. High PRDX1 expression was associated with low tumor grade (*P* <0.001), older age at diagnosis (*P* <0.001) and human epidermal growth factor receptor 2 (Her2) negativity (*P* = 0.001), while it displayed a borderline association with positive ER status (*P* = 0.05) (Table S2 in Additional file 2).

**Figure 2:**
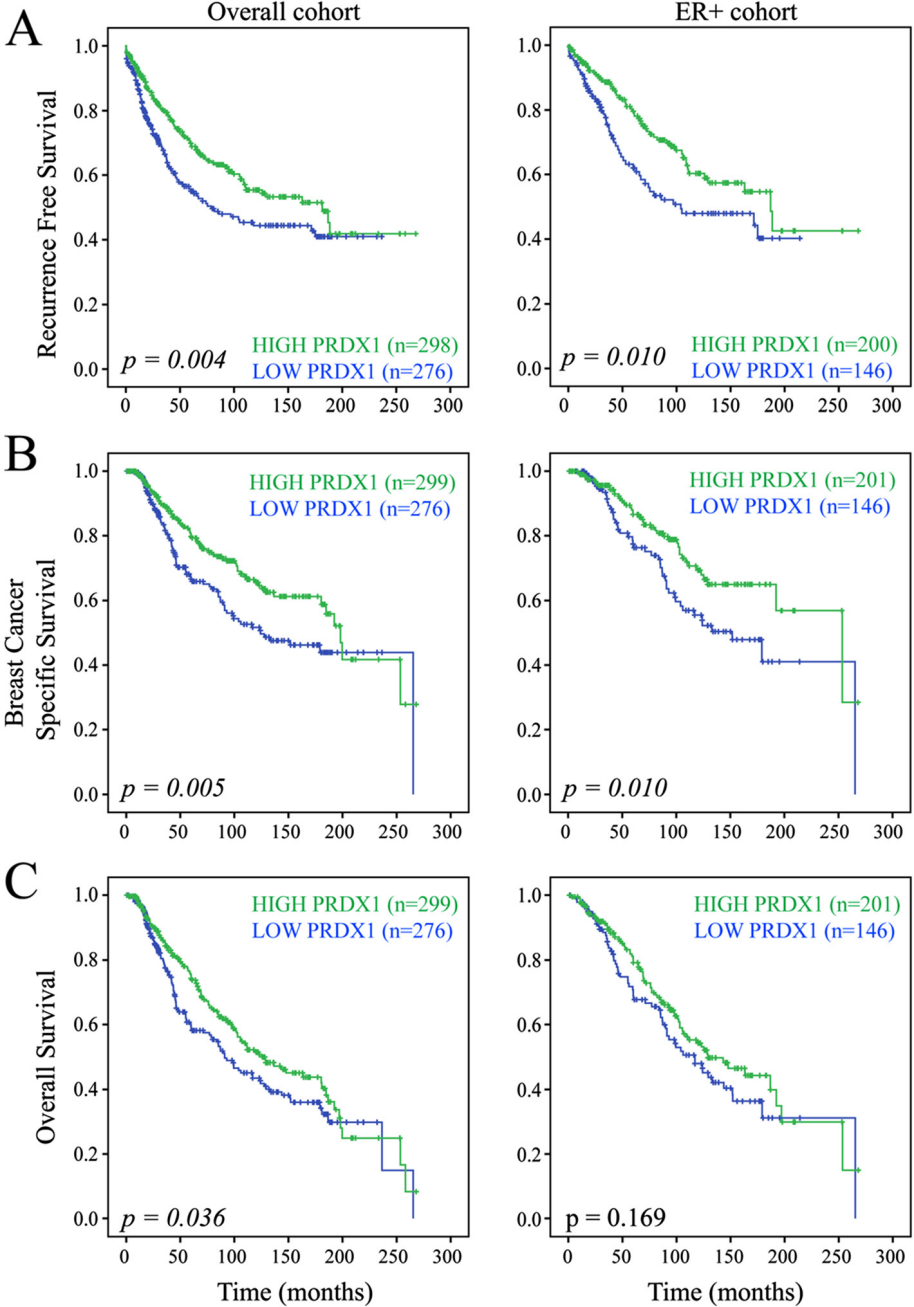
**Prognostic value of PRDX1 protein in ER-positive breast tumors on an RPPA cohort (cohort 1).** Kaplan-Meier analysis showing associations between **(A)** RFS, **(B)** BCSS and **(C)** OS with high/low PRDX1 expression stratified by the median. The overall and ER + cohorts are shown. Significant log-rank *P* values are in *italic* (*P* <0.05). BCSS, breast cancer-specific survival; ER, estrogen receptor; PRDX1, peroxiredoxin 1; OS, overall survival; RFS, recurrence-free survival; RPPA, reverse phase protein arrays.

Kaplan-Meier analysis demonstrated that increased levels of PRDX1 protein expression were associated with improved RFS (*P* = 0.004), OS (*P* = 0.036) and BCSS (*P* = 0.005). Interestingly, when various subcohorts were analyzed, high PRDX1 was associated with improved RFS (*P* = 0.010) and BCSS (*P* = 0.013) only in the subgroup of ER-positive tumors (Figure 2). Univariate Cox regression analysis demonstrated that, in these ER-positive cases, PRDX1 was associated with improved RFS (hazard ratio (HR) = 0.62, 95% confidence interval (CI) = 0.43 to 0.90, *P* = 0.011) and BCSS (HR = 0.59, 95% CI = 0.39 to 0.90, *P* = 0.014) (Table 1). When evaluated by a multivariate Cox proportional hazards model, PRDX1, as assessed by RPPA, was not an independent predictor of RFS or OS (Table 1). However, high PRDX1 protein levels trended toward a significant association with improved BCSS (HR = 0.56, 95% CI = 0.31 to 1.01, *P* = 0.055).

**Table 1:** Cox univariate and multivariate analysis of RFS, BCSS and OS in cohort 1 (RPPA), and also stratified into the ER-positive cohort

	**RFS**		**BCSS**		**OS**	
	**HR (95% CI)**	***P***	**HR (95% CI)**	***P***	**HR (95% CI)**	***P***
**All patients (n = 712)**						
PRDX1 protein	Univariate		Univariate		Univariate	
Low	1		1		1	
High^#^	0.67 (0.52 - 0.88)	*0.004*	0.65 (0.48 - 0.88)	*0.005*	0.76 (0.59 - 0.98)	*0.036*
PRDX1 protein	Multivariate^*^		Multivariate^*^		Multivariate^*^	
Low	1		1		1	
High^#^	0.94 (0.65 - 1.35)	0.720	0.84 (0.55 - 1.28)	0.405	0.93 (0.66 - 1.31)	0.668
**ER + patients (n = 429)**						
PRDX1 protein	Univariate		Univariate		Univariate	
Low	1		1		1	
High^#^	0.62 (0.43 - 0.90)	*0.011*	0.59 (0.39 – 0.90)	*0.014*	0.79 (0.57 - 1.11)	0.171
PRDX1 protein	Multivariate^*^		Multivariate^*^		Multivariate^*^	
Low	1		1		1	
High^#^	0.65 (0.37 - 1.13)	0.129	0.56 (0.31 - 1.01)	0.055	0.78 (0.50 - 1.23)	0.295

^*^Multivariate analysis included adjustment for tumor size, grade, age, nodal, ER, PR and Her2 status. Tumor size and age are both continuous variables. PRDX1 protein expression levels are stratified by the median. ^#^PRDX1 protein expression levels are stratified by the median protein expression value. BCSS, breast cancer-specific survival; CI, confidence interval; ER, estrogen receptor; Her2, human epidermal growth factor receptor 2; HR, hazard ratio; OS, overall survival; PR, progesterone receptor; PRDX1, peroxiredoxin 1; RFS, recurrence-free survival; RPPA, reverse phase protein arrays.

### PRDX1 protein levels, as assessed by IHC, is an independent predictor of prognosis in ER-positive breast tumors

Although RPPA technology is an ideal platform for initial biomarker discovery, it was necessary to validate these findings in an independent cohort using a more clinically applicable platform, namely IHC (cohort 2). Due to core loss during sectioning, tumors from 442 (88.8%) patients were suitable for analysis. IHC staining was scored on a continuous scale based on staining intensity (Figure 1F), and the median expression level was used to stratify the cohort into high and low PRDX1 protein staining. On the basis of this classification, any possible associations between protein expression and a variety of well-defined clinicopathological variables in the TMA cohort were investigated (Table S3 in Additional file 2). PRDX1 protein expression correlated with smaller tumor size (*P* = 0.011), low tumor grade (*P* = 0.002), negative Ki67 status (*P* = 0.004) and lobular subtype (*P* = 0.003), whilst there was a borderline significant correlation with positive ER status (*P* = 0.05).

**Figure 3:**
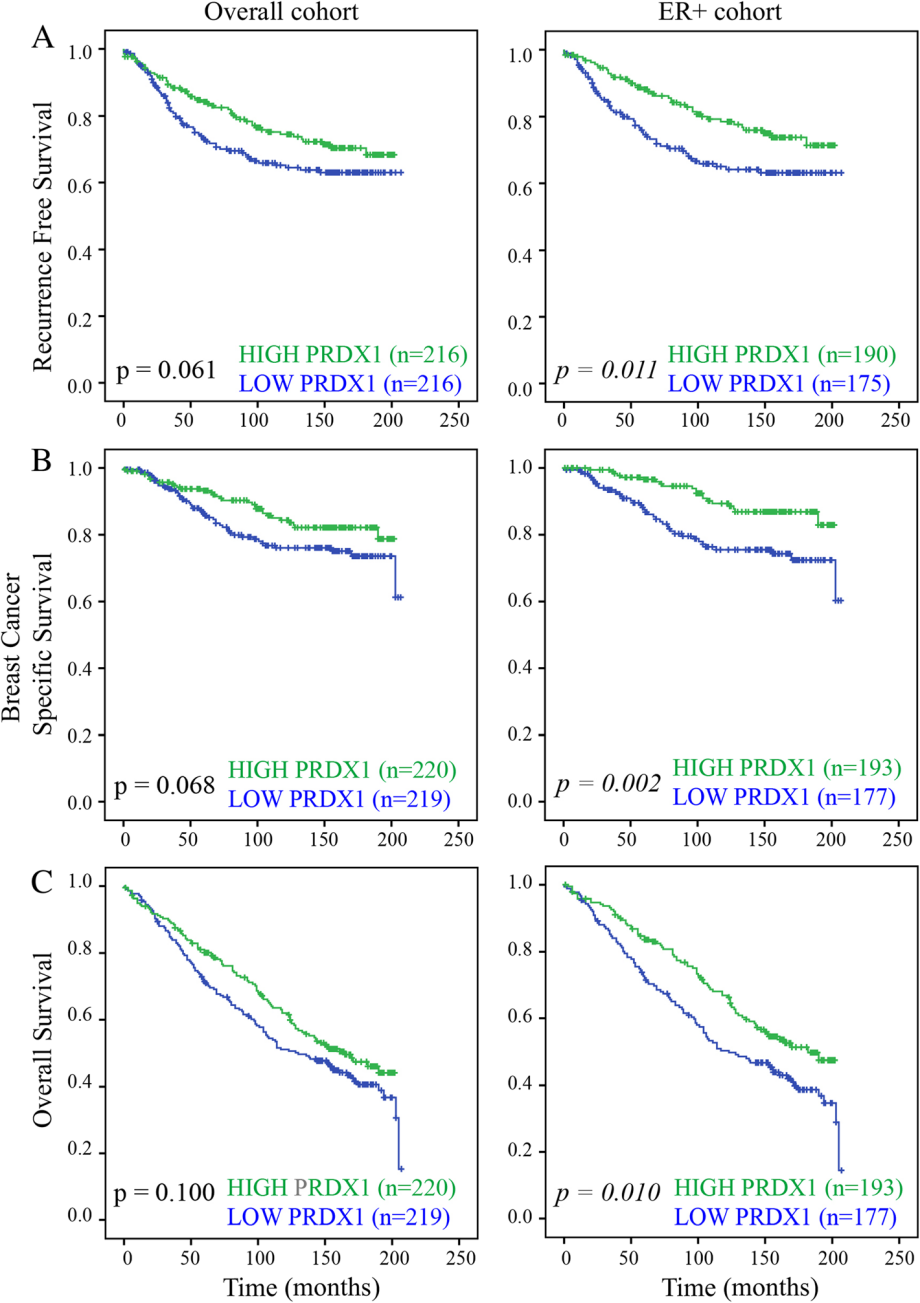
**Prognostic value of PRDX1 protein in primary ER-positive breast tumors on an independent TMA (cohort 2).** Kaplan-Meier analysis showing associations between **(A)** RFS, **(B)** BCSS and **(C)** OS with high/low PRDX1 expression stratified by the median. The overall and ER + cohorts are shown. Significant *P* values are in *italic* (*P* <0.05). BCSS, breast cancer-specific survival; ER, estrogen receptor; OS, overall survival; PRDX1, peroxiredoxin 1; RFS, recurrence-free survival; TMA, tissue microarray.

The relationship between differential expression of PRDX1 and patient survival was subsequently examined. In the overall cohort, increased levels of PRDX1 protein trended toward an improved RFS (*P* = 0.061), OS (*P* = 0.100) and BCSS (*P* = 0.068), but these values were not significant. The positive correlation between high PRDX1 expression and favorable prognosis was again limited to the ER-positive subgroup of cases. Kaplan-Meier analysis demonstrated that, in the ER-positive subgroup, increased levels of PRDX1 protein were associated with an improved RFS (*P* = 0.011), OS (*P* = 0.010) and BCSS (*P* = 0.002) (Figure 3). To compare the prognostic impact of PRDX1 with established factors, Cox regression analysis was performed (Table 2). Univariate Cox regression analysis confirmed that, in ER-positive tumors, high PRDX1 expression associated with improved RFS (HR = 0.60, 95% CI = 0.40 to 0.89, *P* = 0.012), BCSS (HR = 0.69, 95% CI = 0.52 to 0.92, *P* = 0.010) and OS (HR = 0.44, 95% CI = 0.24 to 0.75, *P* = 0.003). Importantly, multivariate analysis within the ER-positive subset showed that PRDX1 was a significant independent predictor of improved RFS (HR = 0.62, 95% CI = 0.40 to 0.96, *P* = 0.032), BCSS (HR = 0.44, 95% CI = 0.24 to 0.79, *P* = 0.006) and OS (HR = 0.61, 95% CI = 0.44 to 0.85, *P* = 0.004), when adjusted for well-established variables such as tumor size, grade, age, nodal, progesterone receptor (PR), Her2 and Ki67 status (Table 2).

**Table 2:** Cox univariate and multivariate analysis of RFS, BCSS and OS in cohort 2 (TMA), and also stratified into the ER-positive cohort

	**RFS**		**BCSS**		**OS**	
	**HR (95% CI)**	***P***	**HR (95% CI)**	***P***	**HR (95% CI)**	***P***
**All patients (n = 432)**						
PRDX1 protein expression	Univariate		Univariate		Univariate	
Low	1		1		1	
High^#^	0.71 (0.50 - 1.02)	0.063	0.81 (0.62 - 1.04)	0.102	0.66 (0.42 - 1.04)	0.071
PRDX1 protein expression	Multivariate^*^		Multivariate^*^		Multivariate^*^	
Low	1		1		1	
High^#^	0.78 (0.52 - 1.16)	0.218	0.76 (0.45 - 1.28)	0.302	0.76 (0.56 - 1.03)	0.076
**ER + patients (n = 365)**						
PRDX1 protein expression	Univariate		Univariate		Univariate	
Low	1		1		1	
High^#^	0.60 (0.40 - 0.89)	*0.012*	0.69 (0.52 - 0.92)	*0.010*	0.44 (0.25 - 0.75)	*0.003*
PRDX1 protein expression	Multivariate^*^		Multivariate^*^		Multivariate^*^	
Low	1		1		1	
High^#^	0.62 (0.40 - 0.96)	*0.032*	0.44 (0.24 - 0.79)	*0.006*	0.61 (0.44 - 0.85)	*0.004*

^*^Multivariate analysis included adjustment for tumor size, grade, age, nodal, ER, PR, Her2 and Ki67 status. PRDX1 protein expression levels stratified by the median. ^#^PRDX1 protein expression levels are stratified by the median protein expression value. BCSS, breast cancer-specific survival; CI, confidence interval; ER, estrogen receptor; Her2, human epidermal growth factor receptor 2; HR, hazard ratio; OS, overall survival; PR, progesterone receptor; PRDX1, peroxiredoxin 1; RFS, recurrence-free survival; RPPA, reverse phase protein arrays.

### Screening for correlations between PRDX1 and cancer-related signaling proteins in clinical breast cancer samples

**Figure 4:**
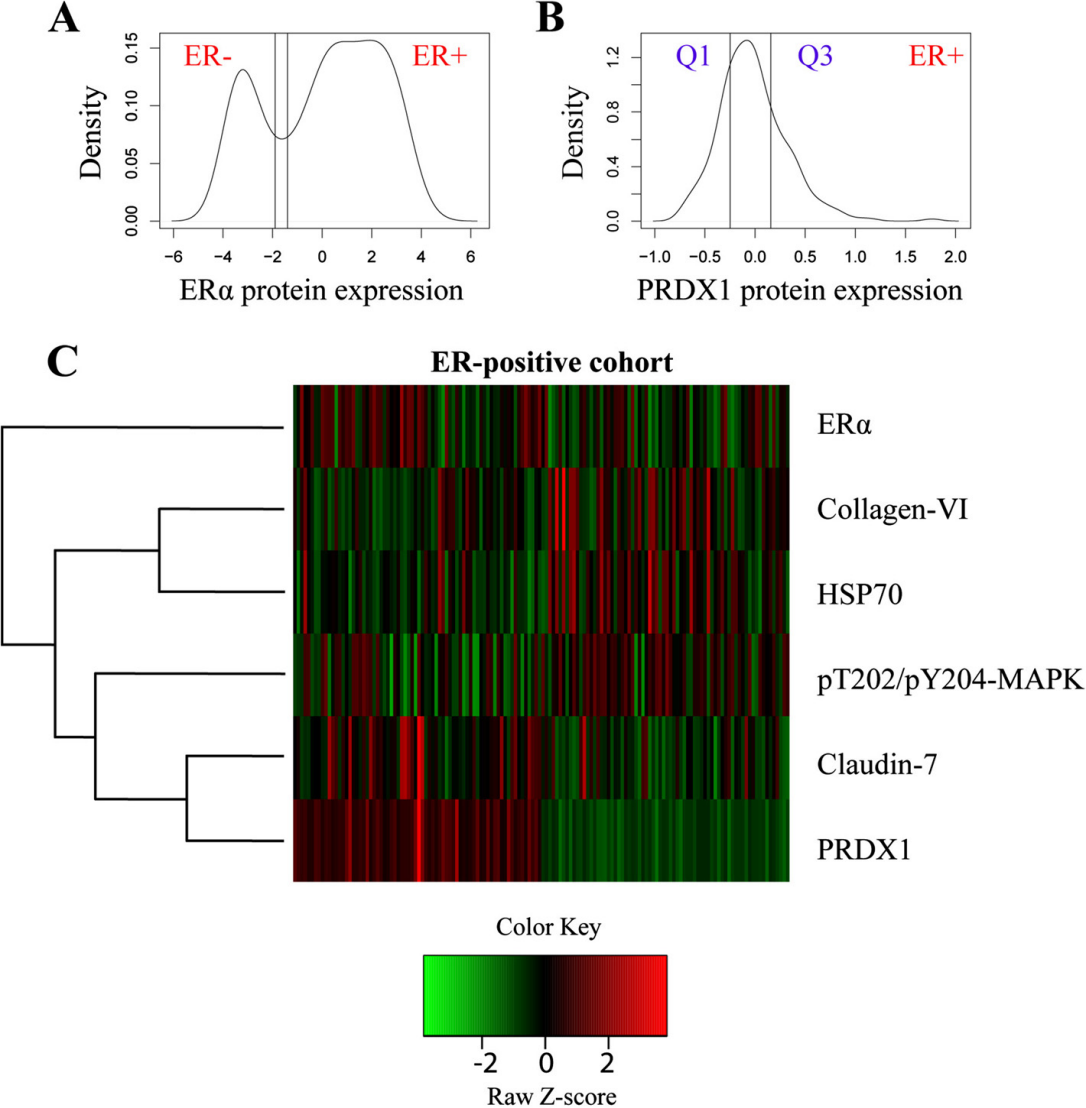
**PRDX1 protein expression and correlation with cancer-signaling proteins in ER-positive tumors from the TCGA RPPA cohort (cohort 3). (A)** ERα protein distribution across all breast tumors as measured by RPPA. The threshold for ER-negative and -positive cases was < -1.9 and > -1.4 of the log_2_ transformed signal, respectively. **(B)** PRDX1 protein distribution across the ER-positive cohort (n = 289). **(C)** Differentially expressed proteins between the upper and lower quartiles of PRDX1 protein expression (quartile size: n = 72) in ER-positive tumors. ERα and Claudin-7 are upregulated while Collagen-VI, pT202/pY204-MAPK and HSP70 are downregulated in PRDX1 high tumors. These proteins are selected based on the adjusted *P* value <0.05 and FC_abs_ <0.67 or >1.5. ER, estrogen receptor; PRDX1, peroxiredoxin 1; RPPA, reverse phase protein arrays; TCGA, The Cancer Genome Atlas.

RPPA technology can be used to explore cellular signaling pathways activated during cancer progression, such as proteins involved in cellular functions such as growth, proliferation and apoptosis [27]. Utilizing an independent cohort of 410 patients (cohort 3), quantitative protein and phosphoprotein expression levels (165 proteins assessed) were screened to identify alterations in response to differential PRDX1 protein expression. This cohort was stratified into ER-positive and ER-negative cohorts based on the quantitative level of ERα protein expression (Figure 4A). This analysis showed a large number of the proteins as significantly correlated with PRDX1 protein expression in the ER-positive cohort (Table S4 in Additional file 2). Thus, a more stringent approach was necessary to refine the proteins altered in response to differential PRDX1 expression levels, whereby proteins altered between the highest quartile (75th percentile and above) versus the lowest quartile (up to 25th percentile) of PRDX1 expression were selected (Figure 4B). A statistically significant positive correlation was seen between PRDX1 protein and ERα (fold change (FC) = 1.54, *P* = 0.017). Other proteins that were altered include Claudin-7 (FC = 1.60, *P* <0.001), HSP70 (FC = 0.61, *P* <0.001), Collagen VI (FC = 0.61, *P* = 0.009) and pThr202/pTyr204-MAPK (FC = 0.65, *P* = 0.008) (Table 3) (represented graphically in Figure 4C). Interestingly, several of the proteins which correlated with PRDX1 expression are known to be regulated by oxidative stress and/or play a role in signaling in the context of ER-positive breast cancer.

**Table 3:** Proteins altered with PRDX1 protein expression in cohort 3 after stratification into the ER-positive cohort

Protein*	**Gene**	**FC**^**#**^	**Log**_**2 **_**FC**	***P *** **value**	**Adjusted *P *** **value**
**ER-positive**					
PRDX1	PRDX1	1.7818	0.8334	<0.0001	*<0.0001*
Claudin-7	CLDN7	1.5971	0.6754	<0.0001	*0.0003*
ER-alpha	ESR1	1.5398	0.6227	0.0040	*0.0169*
MAPK-pT202-Y204	MAPK1 MAPK3	0.6505	-0.6204	0.0011	*0.0075*
Collagen-VI	COL6A1	0.6074	-0.7192	0.0015	*0.0091*
HSP70	HSPA1A	0.6057	-0.7233	0.0000	*0.0007*

^*^Significant adjusted *P* value (*P* <0.05) highlighted in *italics*. ^**#**^FC, fold change. ER, estrogen receptor; PRDX1, peroxiredoxin 1.

### PRDX1 protects against oxidative stress-induced ERα suppression

Since the hypoxic environment of tumors is likely to contain high levels of ROS [31], induction of oxidative stress in cell lines may be a close mimic of intra-tumoral conditions. In addition, oxidative stress is a very well-known modulator of several oncogenic signaling pathways, including the Akt-mediated pathway, where effects of PRDX1 have been already described in mouse mammary tissue [6]. Therefore, we examined whether breast cancer cell lines exposed to oxidative stress had altered levels of these estrogen and breast cancer signaling proteins, and if modulation of PRDX1 expression could alter any oxidative stress-induced signaling changes. Increased oxidative stress was accomplished via treatment with exogenous H_2_O_2_ or peroxynitrite. The ER-positive ductal carcinoma cell line, ZR-75-1, was primarily utilized, being a standard model of ER-positive and estrogen-dependent tumors, with low-level oncogenic signaling [32,33]. In the previous RPPA screen, we observed that ERα and pThr202/pTyr204-MAPK were associated with PRDX1 protein expression in ER-positive tumors (cohort 3). Thus, we set about validating these findings in our model cell line. We observed that H_2_O_2_ treatment resulted in decreased ERα protein expression in parental ZR-75-1 cells with suppression of the ER activity surrogate, PR, as well as enhanced phosphorylation of serine 473 on Akt, another oncogenic signaling molecule (Figure S2A in Additional file 4).

**Figure 5:**
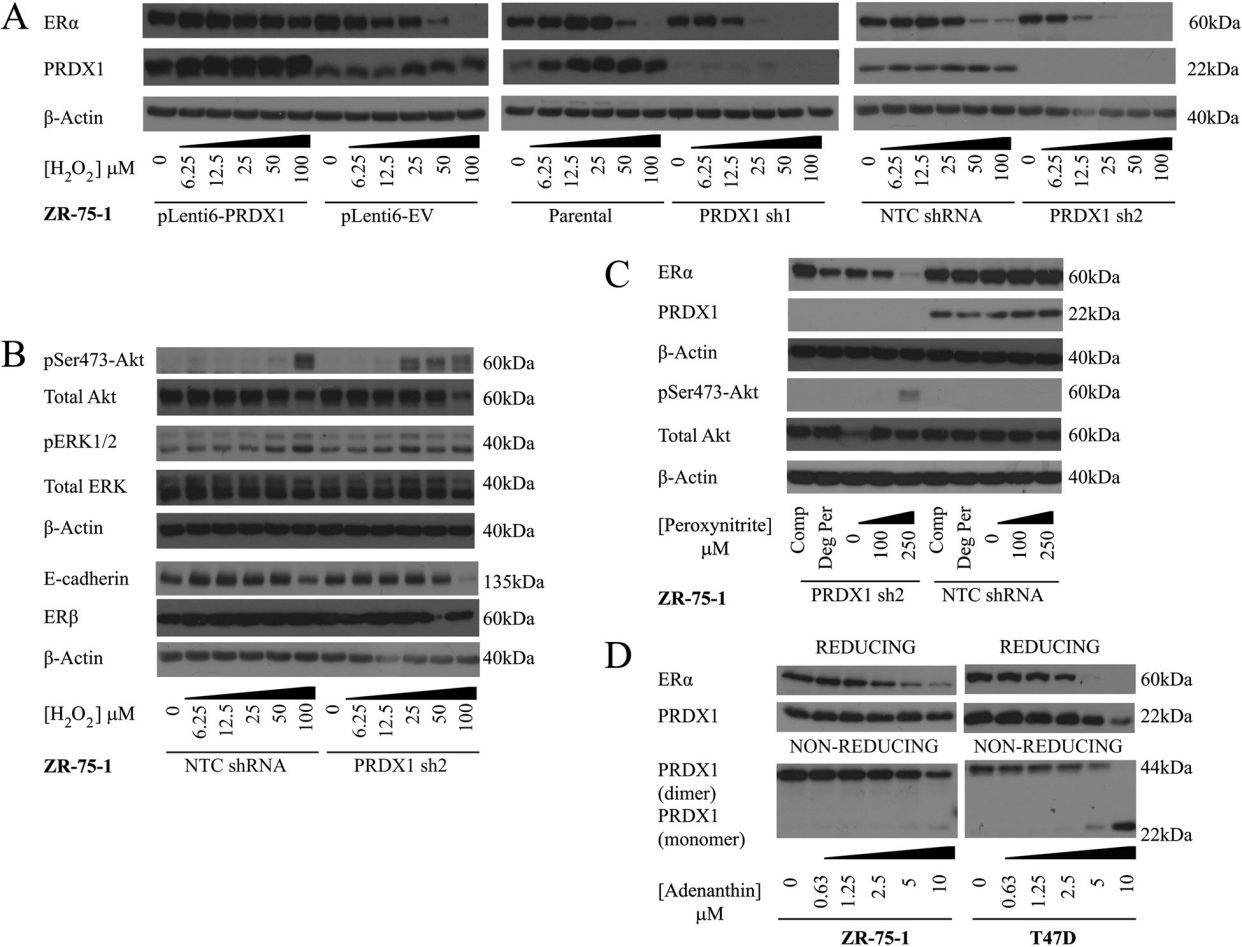
**Effect of PRDX1 knockdown and overexpression on the response of ERα protein expression following induction of oxidative stress in breast cancer cells. (A)** PRDX1 knockdown sensitizes these ER-positive cells to H_2_O_2_-mediated ERα protein suppression while overexpression allows the cells resistance to this suppression. **(B)** PRDX1 knockdown sensitizes these cells to the concurrent phosphorylation of Akt at Serine-473 after treatment with increasing concentrations of H_2_O_2_, while it does not markedly alter expression of E-cadherin or phosphorylation of ERK1/2 after treatment with increasing concentrations of H_2_O_2_ for 16 hours. **(C)** PRDX1 knockdown sensitizes the cells to peroxynitrite-induced suppression of ERα and phosphorylation of Akt. **(D)** Inhibition of the antioxidant activity of PRDX1 using adenanthin suppresses ERα levels in ZR-75-1 and T47D cells, with gradual PRDX1 monomer formation. All treatments were 16 hours in duration. (Comp: complete medium only; DegPer: degraded peroxynitrite). ER, estrogen receptor; H_2_O_2_, hydrogen peroxide; PRDX1, peroxiredoxin 1.

Subsequent studies demonstrated that oxidative stress-mediated suppression of ERα can be regulated by inducing changes in PRDX1 protein expression. In two independently transduced ZR-75-1 cell lines stably expressing different PRDX1-targeting shRNAs, PRDX1 knockdown-enhanced H_2_O_2_-mediated suppression of ERα protein (suppressed at 12.5 μM H_2_O_2_), relative to parental and non-targeting control (NTC) expressing cells (50 μM H_2_O_2_). Conversely, PRDX1 overexpression rendered the cells resistant to suppression of ERα by oxidative stress (Figure 5A). PRDX1 knockdown also enhanced oxidative stress-induced Akt phosphorylation (Ser473) with phosphorylation occurring at a lower H_2_O_2_ concentration (Figure 5B). This effect was also seen when cells were treated with the reactive nitrogen species, peroxynitrite (Figure 5C), which was in accordance with previous observations in other ER-positive breast cancer cell lines [34]. Although H_2_O_2_ induced a decrease of E-cadherin protein expression in breast cancer cells [35] (Figure S2A in Additional file 4), these changes were only minimally affected by changes in PRDX1 expression. Also, H_2_O_2_ induced only marginal or no changes in pThr202/pTyr204-MAPK (ERK1/2) and ERβ expression regardless of PRDX1 expression levels [36,37] (Figure 5). This is consistent with the selectivity of the association between PRDX1 and ER expression. ER activity was also assessed using an estrogen transcriptional response element (ERE)-luciferase reporter assay, whereby PRDX1 knockdown could potentiate oxidative stress-mediated suppression of ERα activity (25 μM H_2_O_2_: *P* = 0.027; 50 μM: *P* = 0.037; 100 μM: *P* = 0.002) (Figure S2B in Additional file 4). In order to determine if PRDX1 expression was driven by ERα promoter activity, we assessed PRDX1 protein expression in ZR-75-1 cells with or without stimulation with the ER ligand, 17β-estradiol. No change in PRDX1 protein content was seen following 17β-estradiol stimulation, and as expected, ERα protein is suppressed upon estradiol stimulation (Figure S2C in Additional file 4). Although *ESR1* mRNA expression is decreased by induction of oxidative stress (Figure S3A in Additional file 5), PRDX1 silencing does not enhance this suppression. Alternatively in PRDX1-silenced ZR-75-1 cells, an *ESR1* induction is seen at lower H_2_O_2_ concentrations, which returns to baseline levels above 50 μM H_2_O_2_. Importantly, Supplementary Figure S3B (Additional file 5) demonstrates that ERα protein levels diminished in these PRDX1-silenced cells across all H_2_O_2_ concentrations used, which suggests that PRDX1 differentially regulates ERα mRNA and protein expression. ZR-75-1 cells were treated with H_2_O_2_, and a transcription inhibitor (actinomycin D) (Figure S3C in Additional file 5) or proteasome inhibitor (MG132) in order to determine if oxidative stress affects ERα protein stability rather than mRNA levels. These results showed that inhibition of proteasomal degradation using MG132 prevents oxidative stress-induced ERα suppression and this inhibition is enhanced in the absence of PRDX1 expression (Figure S3D in Additional file 5), which suggests that the proteasomal degradation of ERα via is more active under low-PRDX1 conditions. This implies that PRDX1 may protect ERα from oxidative stress-induced suppression of the protein itself, rather than transcriptional/mRNA stability regulation. In addition, silencing of PRDX1 in ZR-75-1 and T47D cells did not alter the cell proliferation and apoptosis (Figure S4A-D in Additional file 6).

As expected, PRDX1 shRNA-expressing cells showed a reduced potential to metabolize extracellular H_2_O_2_, similar to the effects of adenanthin, a chemical inhibitor of PRDX1 and PRDX2 antioxidant activity [20] (Figure S2C in Additional file 4). Similar to the effect of exogenous H_2_O_2_ treatment, inhibition of PRDX1 antioxidant activity using adenanthin suppressed ERα levels (Figure 5D; reducing gel). Interestingly, treatment of T47D and ZR-75-1 cell lines with adenanthin resulted in a decrease of PRDX1 dimerization with a dose-dependent increase in PRDX1 monomer levels (Figure 5D; non-reducing gel), which has not been previously reported. Silencing of PRDX1 or PRDX2 diminishes the adenanthin-induced suppression of ERα protein (Figure S5 in Additional file 7). This suggests that PRDX1 and PRDX2 might both have similar and/or partially overlapping effects in protecting ERα from degradation. Importantly for this study, however, adenanthin was initially described as a more potent inhibitor of PRDX1 than PRDX2, as adenanthin had a significantly lower half-maximum inhibitory (IC_50_) values for PRDX1 (1.5 μM) compared to PRDX2 (15 μM) [20]. Indeed, MCF7 cells, which express levels of PRDX1 comparable to ZR-75-1 and T47D, but significantly more PRDX2 protein, require higher concentrations of adenanthin in order to suppress ERα (Figure S5D-E in Additional file 7). This observation suggests that PRDX1 is a primary target for adenanthin, and that PRDX2 can partially substitute for PRDX1-mediated protection of ERα. This potentially opens a new avenue for further investigation of PRDX2 in ER-positive breast cancer.

## Discussion

In order to improve the outcomes for patients, advances in understanding of the pathophysiology of breast cancer combined with the identification of proteins and molecular pathways that affect key proliferation and survival mechanisms are needed. The discovery and validation of these molecular biomarkers requires the integration of several platforms, and antibody-based proteomics allows high-throughput identification and validation of candidate biomarkers (reviewed in [38]).

The aim of this study was to elucidate the association between PRDX1 protein levels and survival in two independent breast cancer cohorts, using two orthologous methods of assessing protein expression levels, namely RPPA and TMA technology. With RPPA, protein lysates are denatured and immobilized to the slide, a similar approach to immunoblotting, and it offers a more quantitative approach for profiling protein expression levels. This proteomic technique has become extremely useful for screening tumor lysates in respect to expression of candidate protein biomarkers. TMAs allow for the IHC-based validation of protein biomarkers, where one could examine proteins in their native non-denatured state and, in particular, to assess spatial pattern of expression which is lost in the RPPA approach. Therefore, both techniques used in this study complement and support each other.

After initially screening a large cohort of breast cancer patients to assess the prognostic potential of PRDX1, we observed that in the ER-positive subset of tumors, high PRDX1 protein expression was associated with improved survival. While RPPA analysis is useful for testing the prognostic ability of biomarkers on limited amounts of tissue, we set about validating these findings using IHC in an independent cohort. Once again, high PRDX1 expression was associated with improved RFS, OS and BCSS in ER-positive patients. In this case, PRDX1 was also an independent predictor of improved survival when adjusted for other clinicopathological variables.

This is the first report showing a functional connection between expression of PRDX1 and ERα in breast cancer. As PRDX1 is a natural antioxidant enzyme, we were interested in further elucidating the links between PRDX1, ROS and regulation of ERα levels in breast cancer. ROS are endogenously produced in all metazoan organisms as a result of aerobic respiration. ROS are essential regulators of cell signaling pathways; however, oxidative stress can occur if ROS production exceeds the capacity of the antioxidant machinery, of which the peroxiredoxin enzymes constitute important members. It has been suggested that the role of oxidative stress in ER-positive breast cancer may be different than in other tumor types [39]. Several studies have demonstrated *in vitro* that mitochondrial ROS can be induced by physiological estrogen concentrations [40,41]. As oxidative metabolism of estrogen and subsequent formation of ROS are key estrogen-related carcinogenic mechanisms [42,43], ROS scavenging systems are expected to play a particularly important role in ER-positive malignancies.

Utilizing an independent cohort of patients (cohort 3), we screened for cancer-related signaling proteins altered in PRDX1 positive tumors. ERα protein is upregulated in PRDX1-high tumors. Although this correlation is of modest potency, it supports our mechanistic observations *in vitro*, as regulation of ERα expression in cells depends on a multiplicity of factors, with PRDX1-mediated protection representing only one. This screening approach also identified other cancer progression-related proteins differentially regulated by PRDX1 in the ER-positive cohort. PRDX1 is positively correlated with the tight junction protein, Claudin-7 [44], while negatively correlated with several proteins involved in malignant cell transformation and epithelial-mesenchymal transition (EMT): the pro-EMT molecule, Collagen VI, [45]; heat shock chaperone protein, HSP-70 [46]; and the phosphorylated form of ERK1/2 (pT202/pY204). These results suggest that PRDX1 may interact with different intracellular ligands within ER-positive tumors, possibly explaining the association of PRDX1 to improved outcomes amongst the ER-positive tumor subtypes. Further studies may elucidate the functional role of the interactions between PRDX1 and these proteins.

Our *in vitro* studies suggest that PRDX1 may play a role as a regulator of the balance between ERα-mediated and oncogene-induced growth patterns in this disease. Specifically, PRDX1 helps to maintain ERα protein levels under oxidative stress, and inhibits the activation of Akt under oxidative stress. This function may be particularly interesting due to the association between PI3K pathway hyperactivity and lower ER levels and activity in ER-positive breast cancer [47]. Although PRDX1 can regulate PTEN [6], the phosphatase that acts upstream of Akt, this mechanism is not relevant in ZR-75-1 cells, as these cells are PTEN-deficient [48]. Our results suggest that PRDX1 protects ERα indirectly through scavenging of H_2_O_2_, as inhibition of antioxidant activity using adenanthin suppresses ERα expression. In addition, the oligomeric state of cellular PRDX1 is a key indicator of PRDX1 function, with dimers primarily acting as an antioxidant scavenger and decameric PRDX1 functioning as a molecular chaperone [49-51]. However, follow-up experiments using *PRDX1* mutants would further tease out this mechanism. *PRDX1* C51/172S mutants are unable to form dimeric the PRDX1 structures required for antioxidant activity [52], while the *PRDX1* mutant (C83S) is incapable of producing decameric PRDX1. Treatment of both *PRDX1* mutant cell lines with H_2_O_2_ would further allow the elucidation of the role of PRDX1 in the protection of ERα. Furthermore, assessment of ERα expression after adenanthin treatment in the presence and absence of H_2_O_2_ could elucidate this mechanism.

Due to high homology between PRDX1 and PRDX2, we also assessed the ability of PRDX1 in maintaining ERα protein levels in absence of PRDX2. This approach showed that both proteins can independently contribute *in vitro* to protecting ERα protein expression. Further *in vivo* studies would elucidate the functional overlap between these two peroxiredoxins in breast cancer.

Recent studies identified an oxidant-sensitive subset of estrogen/ER-responsive breast cancer genes linked to cell growth and invasion pathways that was associated with loss of progesterone receptor and earlier disease-specific mortality [53]. Assuming that oxidative stress contributes to the development of an aggressive subset of primary ER-positive breast cancers, these findings suggest that PRDX1 may be able to protect against these oxidative stress-induced changes in cellular phenotype. It is important to notice that associations with PRDX1 and the endocrine system have been recently described in prostate cancer, including effects of PRDX1 on androgen receptor activity [8,54] and the response to anti-androgen therapies [55]. Along with our report, this suggests a key role for PRDX1 in regulation of the activity of steroid hormone-related pathways. Interestingly, it has been suggested that resistance to endocrine therapy may be mediated, in part, by ROS-mediated dysregulation of estrogen signaling pathways (reviewed in [56]).

Previous studies on PRDX1 expression did not find an association with clinicopathological features or prognosis in human breast cancer [12,13]. Potentially, the antibodies used by these studies may lack specificity for PRDX1 or alternatively, in contrast to the large sample sets studied herein, insufficient sample numbers and power may have precluded detection of associations with outcomes. Significantly, one of the above mentioned studies reports a strong nuclear pattern of PRDX1 expression as determined by IHC staining (Figure 1A in [13]), compared to our observation where PRDX1 is predominantly cytoplasmic. This discrepancy underscores the need for rigorous testing of antibodies in biomarker studies. Our antibody validation model includes testing using recombinant cell lines and tumor tissues across several platforms of protein quantification (immunoblotting, RPPA, cell pellet arrays, TMAs). Altogether, this study provides a model to accelerate the validation of potential biomarkers on the translational journey to the clinic.

## Conclusions

In conclusion, we have identified PRDX1 protein as an independent predictor of favorable prognosis in ER-positive breast carcinomas. Based on our accumulated data, we hypothesize that PRDX1 shields the dependence of mammary tumors on estrogen-mediated growth stimulation, which eventually is of benefit for the patient. In this regard, PRDX1 expression may be utilized in the therapeutic decision-making process in this disease in the future. Moreover, our results suggest that any prospective PRDX1 inhibitors should be explored with caution in ER-positive breast cancer due to the potential to convert tumor cells to a more aggressive phenotype with a worsened outcome.

## Abbreviations

ABTS: 2,2’-azino-bis(3-ethylbenzothiazoline-sulfonic acid) diammonium salt solution; API: average positive intensity; BCA: bicinchoninic acid; BCSS: breast cancer-specific survival; CI: confidence interval; DAB: 3,3-diaminobenzidine; DMEM: Dulbecco’s modified Eagle’s medium; EMT: epithelial-mesenchymal transition; ER: estrogen receptor; ERE: estrogen transcriptional response element; FBS: fetal bovine serum; FC: fold change; FFPE: formalin-fixed paraffin-embedded; GFP: green fluorescent protein; H_2_O_2_: hydrogen peroxide; Her2: human epidermal growth factor receptor 2; HPA: Human Protein Atlas; HR: hazard ratio; HRP: horseradish peroxidase; IHC: immunohistochemistry; MDACC: M. D. Anderson Cancer Center; NTC: non-targeting control; OS: overall survival; PBS-T: phosphate-buffered saline with 0.1% Tween-20; PR: progesterone receptor; PRDX1: peroxiredoxin 1; qPCR: quantitative PCR; RFS: recurrence-free survival; RIPA: radioimmunoprecipitation buffer; ROS: reactive oxygen species; RPPA: reverse phase protein arrays; RT: room temperature; RT-PCR: real-time polymerase chain reaction; SDS: sodium dodecyl sulfate; shRNA: short hairpin loop RNA; TBST: tris-buffered saline with 0.1% Tween-20; TCGA: The Cancer Genome Atlas; TMA: tissue microarray.

## Competing interests

No potential conflicts of interest were disclosed.

## Authors’ contributions

PCOL generated the PRDX1 recombinant cell lines, carried out the PRDX1 molecular genetic studies, antibody validation, immunostaining and analysis of the cohort 2 TMA and cohort 3 RPPA, survival analysis for all cohorts, and drafted the initial copy of the manuscript. RZ, MT and MB generated the PRDX2-silenced cell lines and carried out the RT-PCR, WB and apoptosis analysis. MT carried out the adenanthin treatment study on PRDX2-silenced lines. BTH, JL, AMG and GBM participated in the collection of survival data, slide preparation, immunostaining and analysis of the cohort 1 RPPA. KC contributed to the Western blotting experiments for the manuscript revision. KJ collected all patient data and prepared the TMA for cohort 2. FP and MU provided the PRDX1 antibody and participated in initial antibody validation. PCOL, PG, MT, GBM and RZ participated in the statistical analysis of the data for cohort 3. AZ, RZ and MB produced the PRDX1 and PRDX2 overexpression vectors. HDS and JXP carried out the isolation and synthesis of the adenanthin compound. PCOL, DPOC, DJB, DN, JPC, MT, RZ and WMG were involved in the conception and design of the study. RZ and WMG participated in its coordination and helped to draft the manuscript. All authors critically revised the initial draft of the manuscript and subsequent revisions. All authors approved the manuscript in its current form.

## Supplementary Material

Additional file 1 **Supplementary Methods.** Additional methods not described in the main body of text. Click here for file

Additional file 2: Table S1 Clinicopathological parameters for the three cohorts of breast cancer patients utilized in this study. **Table S2.** Association of PRDX1 expression with clinicopathological parameters in the RPPA cohort (cohort 1). **Table S3.** Association of PRDX1 expression with clinicopathological parameters in the consecutive cohort (cohort 2). **Table S4.** Proteins that correlate with PRDX1 protein expression in the ER-positive and/or ER-negative cohort. ER, estrogen receptor; PRDX1, peroxiredoxin 1; RPPA, reverse phase protein arrays. Click here for file

Additional file 3: Figure S1 Additional antibody validation experiments. **(A)** Full length immunoblotting gel shows a single discrete signal, the intensity of which correlates with PRDX1 knockdown/overexpression across recombinant ZR-75-1 cell lines (V5-tagged PRDX1 protein runs at 25 kDa, with native protein at 22 kDa). **(B)** Immunoblotting signal in PRDX1-silenced ZR-75-1 cell lines using independent PRDX1-targeting antibodies. **(C)** Protein and transcript expression were compared across three recombinant ZR-75-1 cells lines (NTC shRNA, PRDX1-sh1, PRDX1-sh2), which shows a greater correlation using the antibody from Atlas antibodies (solid line) compared to the Abcam antibody (dotted line). **(D)** Overexpression of PRDX1 in ZR-75-1 cells did not affect PRDX2 protein expression, while PRDX2 overexpression in T47D cells did not affect PRDX1 protein expression. NTC, non-targeting control; PRDX1, peroxiredoxin 1; shRNA, short hairpin loop RNA. Click here for file

Additional file 4: Figure S2 Additional *in vitro* Western blotting and functional assays. **(A)** Effect of H_2_O_2_ on ERα, pSer473-Akt and E-cadherin levels, along with respective loading controls. Parental ZR-75-1 cells were treated with increasing levels of H_2_O_2_ for 16 hours. **(B)** PRDX1 knockdown enhances the H_2_O_2_-mediated suppression of ER activity. Relative units of the ERE-luciferase reporter expression were normalized to Renilla-luciferase units. H_2_O_2_ treatment was 16 hours in duration. **(C)** PRDX1 protein expression is not driven by ER activity. Western blot analysis shows that no change in PRDX1 protein expression following stimulation of parental ZR-75-1 cells with 1 or 10 nM 17-β estradiol for 48 hours. During this experiment, these cells were cultured in DMEM containing 0.1% FBS supplemented with 17-β estradiol or vehicle control. **(D)** Treatment with adenanthin inhibits metabolism of H_2_O_2_ following three hours treatment with 50 μM H_2_O_2_. Knockdown of PRDX1 (sh2) is shown as a positive control. (ns: non-significant; ^*^P <0.05; ^**^P <0.01). DMEM, Dulbecco’s modified Eagle’s medium; ER, estrogen receptor; ERE, estrogen transcriptional response element; FBS, fetal bovine serum; H_2_O_2_, hydrogen peroxide; PRDX1, peroxiredoxin 1. Click here for file

Additional file 5: Figure S3 Effects on mRNA expression and protein stability after H_2_O_2_ treatment. **(A)***ESR1* mRNA expression is suppressed after induction of oxidative stress (16 hours H_2_O_2_ treatment) in ZR-75-1 cells. Silencing of PRDX1 does not enhance this oxidative stress-mediated suppression of *ESR1* mRNA. Error bars represent the SEM from independent biological experiments (^*^P <0.05; ^**^P <0.01). **(B)** H_2_O_2_ treatment suppresses ERα protein expression in these PRDX1-silenced ZR-75-1 cells. **(C)** Inhibition of transcription (1 μg/μl Actinomycin D) does not alter H_2_O_2_-mediated suppression of ERα protein. **(D)** Inhibition of proteasomal degradation (5 μM MG132) prevents oxidative stress-induced ERα suppression in ZR-75-1 cells. After inhibition of proteasomal degradation, PRDX1-sh2 expressing cells display a weaker H_2_O_2_-mediated suppression. All treatments were 16 hours in duration. ER, estrogen receptor; H_2_O_2_, hydrogen peroxide; PRDX1, peroxiredoxin 1. Click here for file

Additional file 6: Figure S4 Viability and apoptosis assays. PRDX1 knockdown does not alter cell growth or viability in ZR-75-1 and T47D breast cancer cell lines. ZR-75-1 **(A)** and T47D **(B)** cells were grown in normal medium for up to 120 hours, with cell viability (MTT assay) being measured every 24 hours. Experiments were repeated three times with six wells per experiment. Error bars represent the SEM. **(C)** Flow cytometry viability analysis (7-AAD viability staining solution) was also used to demonstrate this lack of change in proliferation. **(D)** The Apotox-Glo™ Triplex assay kit was used to measure caspase 3/7 activity in PRDX1 or PRDX2-silenced ZR-75-1 cells compared to the parental and NTC shRNA controls. Error bars represent the SD (ns: non-significant). PRDX1, peroxiredoxin 1. Click here for file

Additional file 7: Figure S5 PRDX2 RT-PCR and Western blotting, including adenanthin treatment of PRDX2-silenced cells. **(A)** PRDX2 mRNA and **(B)** protein expression is reduced in ZR-75-1 and T47D cells after lentiviral transduction of up to five different anti-PRDX2 shRNAs. **(C)** Silencing of PRDX1 or PRDX2 abrogates the adenanthin-induced suppression of ERα protein in ZR-75-1. All treatments were 16 hours in duration. Error bars represent the SEM from two independent experiments (^***^P <0.001). ER, estrogen receptor; PRDX1, peroxiredoxin 1; RT-PCR, real-time polymerase chain reaction; shRNA, short hairpin loop RNA. Click here for file
