# Supplementary material for: Peroxiredoxin-1 protects estrogen receptor α from oxidative stress-induced suppression and is a protein biomarker of favorable prognosis in breast cancer
Source: Breast Cancer Res. 2014 Jul 10;16(4):R79. doi: 10.1186/bcr3691 (PMC4226972; doi:10.1186/bcr3691)
Supplement: Additional file 1 — Supplementary Methods. Additional methods not described in the main body of text. [file bcr3691-S1.pdf]

Supplementary Methods

Lentiviral overexpression and knockdown of PRDX1

A PRDX1 cDNA-containing plasmid (Clone ID: HsCD00042169) was obtained from the Dana-Farber/Harvard Cancer Center DNA Resource Core (Harvard Medical School, Boston, MA). PRDX1 cDNA was transferred into a pLenti6/V5-DEST vector via use of Gateway cloning system followed by LR Clonase II-mediated reaction (all reagents from Invitrogen, Carlsbad, CA). The PRDX2 cDNA was obtained from Addgene (plasmid clone number 31338). The PRDX2 cDNA was extracted by PCR technique using the following primers: forward 5'-CACCATGGCCTCCGGTAAC-3' and reverse 5'-ATTGTGTTTGGAGAAATATTCCTTGCTGTCATC-3' and using the Phusion polymerase kit. The PCR product was then cloned into pLenti6 lentiviral vector using the ViraPower Lentiviral Directional TOPO Expression Kit (Invitrogen) according to manufacturer's instructions.

Two commercially available (Open Biosystems, Huntsville, AL) GIPZ vector-based short hairpin (sh)RNA lentiviral constructs targeting PRDX1, five commercially available (Open Biosystems) GIPZ vector-based short hairpin (sh)RNA lentiviral constructs targeting PRDX2, or a construct encoding a non-targeted control shRNA were used to make stably transduced cell lines:

- PRDX1 shRNA 1 sequence: ATCACTGCCAGGTTTCCAG
- PRDX1 shRNA 2 sequence: TTCCTTTGTAGTCAGACAG
- PRDX2 shRNA 1 sequence: TGGGCTTAATCGTGTCACT
- PRDX2 shRNA 2 sequence: AATCATTAACAGTGATCTG
- PRDX2 shRNA 3 sequence: AGTCCGACAGCTTCACCTC
- PRDX2 shRNA 4 sequence: AATCCTCAGACAAGCGTCT
- PRDX2 shRNA 5 sequence: TCCTTGCTGTCATCCACGT

Human embryonic kidney (HEK)-293T cells, at 60-70% confluence, were transfected using the Genejuice (Merck Biosciences Ltd, Nottingham, UK) transfection method with the respective plasmids, together with packaging and envelope plasmids (PSPAX2 and PMD2G; courtesy of the Trono Laboratory). The media was refreshed after 8 hours and, after a further 48 hours, the viral supernatant was removed and filtered through a 0.45 μm low-protein binding filter (Millipore, Billerica, MA, USA). This was added to 50% confluent target cells (ZR-75-1, T47D or SKRB3) at a 1:1 dilution with fresh media. Media was refreshed after 24 hours. Additionally, FACS sorting based on GFP fluorescence was also used to further

enhance the shRNA-mediated knockdown by selecting the cells with the highest expression of the GFP-positive vector.

Immunoblotting

For Western blotting analysis, cell lines were lysed using RIPA buffer with protease inhibitor cocktail (Roche, Basel, Switzerland), followed by centrifugation at 20,817 x g (14,000 rpm) at 4°C. The supernatants were removed and the protein concentrations determined by the bicinchoninic acid (BCA) method (Pierce, IL, USA). Protein samples were separated on SDS-PAGE gels and transferred to a PVDF membrane. Membranes were then incubated for 1 hour at room temperature (RT) in 5% low-fat dry milk or bovine serum albumin in 1% TBS-Tween 20 (TBST). After a 4°C overnight incubation in the primary antibody (1:500 anti-PRDX1 (Atlas Antibodies, Stockholm, Sweden); 1:2000 anti-PRDX2 (AbFrontier, South Korea); 1:250 anti-ERα-6F11, anti-ERβ 1:500 (Novacastra/Leica, Wetzlar, Germany); 1:1000 anti-pSer473-Akt, 1:1000 anti-total Akt, 1:1000 anti-E-cadherin (Cell Signaling, Beverly, MA, USA); 1:500 anti-pERK1/2, 1:500 anti-total ERK1/2 (Santa Cruz Biotechnology, Santa Cruz, CA, USA); 1:2500 anti-V5 (Invitrogen); 1:20000 anti-β-actin (Sigma)), the cells were washed thrice for 10 minutes in TBS-T, incubated for 1 hour at RT with a horseradish peroxidase-conjugated secondary antibody, and again washed thrice in TBST. The proteins were detected using Enhanced Chemiluminescence plus (Amersham Biosciences, UK). Chemiluminescence was detected by autoradiography using X-ray film (Fujifilm, Japan).

Reverse phase protein array analysis

Proteins were extracted from tissue or cell lines using RPPA lysis buffer (1% Triton X-100, 50 mM HEPES, pH 7.4, 150 mM NaCl, 1.5 mM $MgCl_2$, 1 mM EGTA, 100 mM NaF, 10 mM Na pyrophosphate, 1 mM Na_3VO_4, 10% glycerol, containing freshly added protease and phosphatase inhibitors (Roche)). Samples were centrifuged at 20,817 x g (14,000 rpm) at 4°C. The supernatants were removed and the protein concentrations determined by the bicinchoninic acid (BCA) method (Pierce, IL, USA) and adjusted by diluting in lysis buffer to 1-1.5mg/ml.

Cellular proteins were denatured by 1% SDS (with beta-mercaptoethanol) and diluted in five 2-fold serial dilutions in dilution buffer (lysis buffer containing 1% SDS). Serial diluted lysates were arrayed on nitrocellulose-coated FAST slides (Whatman) using the Aushon 2470

Arrayer (Aushon Biosystems). A total of 5808 array spots were arranged on each slide, including the spots corresponding to positive and negative controls prepared from mixed cell lysates or dilution buffer, respectively.

Each slide was probed with a validated primary antibody plus a biotin-conjugated secondary antibody. Only antibodies which generated a Pearson correlation coefficient (r) between RPPA and Western blotting densitometry data of greater than 0.7 in respect to cell culture models were used in RPPA-based analysis of tissue extracts. In more detail, antibodies which provided a discrete signal via Western blotting were further assessed by direct comparison to RPPA using cell lines with a dynamic range of protein expression, using protein lysates from recombinant cell lines with overexpression or knockdown of the protein-of-interest. The signal obtained was amplified using a Dako Cytomation–catalyzed system (Dako) and visualized by a DAB colorimetric reaction. The slides were scanned, analyzed, and quantified using a customized-software Microvigene (VigeneTech Inc.) to generate spot intensity measurements.

Each dilution curve was fitted with a logistic model ("Supercurve Fitting" developed by the Department of Bioinformatics and Computational Biology at the MD Anderson Cancer Center, "http://bioinformatics.mdanderson.org/OOMPA"). This fits a single curve using all the samples (i.e. dilution series) on a slide with the signal intensity as the response variable and the dilution steps as the independent variable. The fitted curve is plotted with the signal intensities – both observed and fitted - on the y-axis and the log2-concentration of proteins on the x-axis. The protein concentrations of each set of slides were then normalized by median polish, which was corrected across samples by the linear expression values using the median expression levels of all antibody experiments to calculate a loading correction factor for each sample.

Immunohistochemistry

To construct cell pellet arrays, cells were trypsinized and fixed for 10 hours in 10% formalin (Sigma) following which they were centrifuged, washed with phosphate buffered saline (PBS) (Thermo Scientific) and resuspended in 1% agarose (Pronadisa). The tumor cell-containing agarose plugs were processed through gradient alcohols before being cleared in xylene and washed in molten paraffin. These cell pellets were embedded in paraffin and arrayed in quadruplicate 1.0mm cores using a manual tissue arrayer (MTA-1, Beecher Inc,

WI). IHC was carried out on 5µm sections. Tissue microarrays (TMA) were constructed as previously described [1].

Cell pellet arrays or tissue microarrays were deparaffinized in xylene and rehydrated in descending gradient alcohols. Heat-mediated antigen retrieval was performed using 10mM sodium citrate buffer (pH 6) using a PT module (LabVision, UK) for 15 minutes at 95°C. The Ultravision ONE Detection System HRP Polymer & DAB Plus Chromagen kit was used for staining. The slides were incubated with 3% H_2O_2 for 10 minutes, UV blocking agent for 10 minutes, and the anti-PRDX1 antibody (1:150) for 1 hour. Sections were washed in PBS with 0.1% Tween 20 (PBS-T), following which primary antibody enhancer was applied for 20 min, and sections were washed in PBS-T again. Sections were then incubated with HRP polymer for 15 min, washed in PBS-T and then developed for 10 min using diaminobenzidine (DAB) solution (LabVision, UK). All the incubations and washing stages were carried out at room temperature. The sections were counterstained in haematoxylin, dehydrated in alcohol and xylene and mounted using an automated coverslipper (Leica, Germany). As a negative control, the anti-PRDX1 antibody was substituted with an anti-rabbit immunoglobulin G isotype control and with a PBST only negative control.

TCGA (cohort 3) data download and analysis approach

PRDX1 protein expression as determined by RPPA analysis and clinical follow-up data were downloaded directly from the TCGA data portal (https://tcga-data.nci.nih.gov/tcga/). After merging the data based on patient ID number, the cohort was separated into ER-negative and -positive subgroups using expression levels of ERα protein as determined by RPPA. A threshold of < -1.8 and > -1.4 of the log2 transformed signal was used to stratify the ER-negative and -positive subgroups, respectively. Pearson's correlation test was used to check for proteins correlating with PRDX1 protein or mRNA expression levels. The two-sided t-test was used to identify proteins co-regulated between the lower and upper quartile of PRDX1 protein expression cases, using a fold change threshold of 0.67 < FC < 1.5. A p-value of less than 0.05 was considered significant.

Dual-luciferase reporter assays

Cells were transiently transfected with an ERE-Luc reporter construct together with a CMV-Renilla luciferase construct (internal control) using Genejuice reagent (Merck, Darmstadt,

Germany) according to the manufacturer's recommendations. After 48 hours, the cells were treated with H_2O_2 for 16 hours. Relative expression of the ERE-Luc reporter normalized to Renilla Luciferase levels was measured using the Dual Luciferase Reporter Assay kit (Promega, Madison, WI, USA) and a Glomax microplate luminometer with dual injector system (Promega).

Peroxide metabolism assay

Cells were cultured in triplicate in 96 well plates at 20,000 cells/well, rinsed with 1x PBS (37°C), pre-treated with different concentrations of adenanthin and treated with 50 μM H_2O_2 for 3 hours at 37°C. A standard H_2O_2 curve was also carried out in PBS that included various concentrations and incubated for the same period as the cells. The H_2O_2 remaining after 3 hours was measured by transferring 100 μl of sample and standard from each well to a fresh 96 well plate with 25 μl of mixture containing 1 μl HRP (50U/ml in 0.1% Tween 20, 50% glycerol, 0.1% BSA in PBS) and 24 μl 2,2'-azino-bis(3-ethylbenzothiazoline-sulfonic acid) diammonium salt solution (ABTS), and subsequent incubation for 10 min at room temperature followed by reading at 405 nm using a SpectraMax M2 microplate reader (Molecular Devices Corp., Sunnyvale, CA).

Peroxynitrite treatment

Peroxynitrite treatment was for 16 hours in duration. Peroxynitrite is not stable in FBS conditions, so all peroxynitrite treatments were carried out in phenol-red free media with 5% charcoal dextran FBS. Degraded peroxynitrite was used as a control, along with complete media (DMEM + 10% FBS).

RNA extraction, cDNA synthesis and qPCR

For H_2O_2 experiments, ZR-75-1 parental, shNTC and PRDX1 sh2 cells (5×10^5) were cultured for 24 h and treated with different concentrations of H_2O_2 (0, 25, 50, 100 μM) for 16 hours at 37°C. For PRDX2 experiments, ZR-75-1 parental, shNTC and PRDX2 sh1 and PRDX2 sh2 cells (6×10^5) were cultured overnight in 6-well plates. Cells were then collected and total cellular RNA was extracted with Trizol (Invitrogen) or TRIsure™ (Bioline). 1 ug of RNA was used for synthesis of cDNA, for use as a template for quantitative PCR (qPCR).

LightCycler® 480 SYBR Green I Master mix (Roche Applied Science) or Superscript II reverse transcriptase (200 U/µL Invitrogen) were used for the real-time PCR (RT-PCR) reaction. Primers used include ERα forward (5'-TTACTGACCAACCTGGCAGA-3'), ERα reverse (5'-ATCATGGAGGGTCAAATCCA-3') primers, PRDX2 forward (5'-GCCTTCCAGTACACAGACGA-3') and PRDX2 reverse (5'-GTTGGGCTTAATCGTGTCACT-3'); along with β-2 microglobulin and RPL29 primers for use as internal controls. All qPCR reactions were conducted at 95°C for 5 min (pre-incubation), and then 45 cycles of amplification, 95°C for 10 s, 60°C for 10 s and 72°C for 10 s. The specificity of the reaction was verified by melt curve analysis. Experiments were performed using a LightCycler® 480 System (Roche Applied Science) and data processing was performed using LightCycler® 480 software, Version 1.5 (Roche Applied Science) or a 7900HT Fast Real Time PCR System (Applied Biosystems, Foster City, CA, USA), depending on the master mix used.

Flow cytometry

ZR-75-1 shNTC and PRDX1 sh2 cells (1 x 10^6) were cultured overnight onto 25 cm^2 flask in DMEM medium containing 10% FBS (Sigma), 100 U/mL penicillin, 100 µg/mL streptomycin (Gibco) and 1.46 mg/mL L-glutamine, 1 nM β-estradiol without sodium pyruvate. Cells were harvested and washed in cold 1x PBS. The percentage of non-viable cells was assessed by flow cytometry using the 7-AAD Viability Staining Solution (eBioscience, Affymetrix) according to the manufacturer's protocol. Briefly, ZR-75-1 shNTC and PRDX1-sh2 cells were incubated with 5 µl 7-AAD in 200 µl of 1 × binding buffer for 15 min at room temperature protected from light and analyzed by flow cytometry. Experiments were performed using a FACS Aria III cell sorter (BD Bioscience) and data processing was performed using BD FACSDiva™ Software (BD Bioscience).

Apoptosis Assay

A total of 3×10^3 cells were seeded per 384-well and the cells were allowed to adhere overnight. Caspase 3/7 activity was measured after 1 hour incubations, using the Apotox-Glo™ Triplex assay kit and the GloMax®-Multi Microplate Multimode Reader (kits and equipment from Promega). Treatments were carried out using triplicate wells in each biological replicate.

References

1. Kononen J, Bubendorf L, Kallioniemi A, Barlund M, Schraml P, Leighton S, Torhorst J, Mihatsch MJ, Sauter G, Kallioniemi OP: **Tissue microarrays for high-throughput molecular profiling of tumor specimens**. *Nat Med* 1998, **4**(7):844-847.
