# Supplementary material for: Peroxiredoxin-1 protects estrogen receptor α from oxidative stress-induced suppression and is a protein biomarker of favorable prognosis in breast cancer
Source: Breast Cancer Res. 2014 Jul 10;16(4):R79. doi: 10.1186/bcr3691 (PMC4226972; doi:10.1186/bcr3691)
Supplement: Additional file 2: Table S1 — Clinicopathological parameters for the three cohorts of breast cancer patients utilized in this study. Table S2. Association of PRDX1 expression with clinicopathological parameters in the RPPA cohort (cohort 1). Table S3. Association of PRDX1 expression with clinicopathological parameters in the consecutive cohort (cohort 2). Table S4. Proteins that correlate with PRDX1 protein expression in the ER-positive and/or ER-negative cohort. ER, estrogen receptor; PRDX1, peroxiredoxin 1; RPPA, reverse phase protein arrays. [file bcr3691-S2.pdf]

Supplementary Table S1: Clinico-pathological parameters for the three cohorts of breast cancer patients utilized in this study

Variables	Cohort 1<br>Houston RPPA<br>(n = 712)	Cohort 2<br>Consecutive<br>TMA<br>(n = 512)	Cohort 3<br>TCGA RPPA<br>(n = 410)
**Age at Diagnosis**			
Median (Range)	60 (23 - 89)	65 (27 - 96)	58 (26 - 89)
Missing	136 (19.1%)	-	-
**Survival Data**			
Available	575 (80.8%)	439 (85.7%)	-
Missing	137 (19.2%)	73 (14.3%)	-
**Tumor Size**			
≤2cm	179 (25.1%)	267 (60.4%)	-
>2cm	246 (34.6%)	175 (39.6%)	-
Missing	287 (40.3%)	-	-
**Tumor Grade**			
I	65 (9.1%)	107 (24.2%)	-
II	149 (20.9%)	180 (40.7%)	-
III	243 (34.1%)	154 (34.8%)	-
Missing	255 (35.8%)	1 (0.2%)	-
**Nodal status**			
N0	283 (39.7%)	246 (55.7%)	161 (39.3%)
N1+	255 (35.8%)	148 (33.5%)	171 (41.7%)
Missing	174 (24.4%)	48 (10.9%)	78 (19.0%)
**ER status***			
ER negative	262 (36.8%)	65 (14.7%)	99 (24.1%)
ER positive	429 (60.3%)	353 (79.9%)	304 (74.1%)
Missing	21 (2.9%)	24 (5.4%)	7 (1.7%)
**PR status**			
PR negative	367 (51.5%)	125 (28.3%)	156 (38.0%)
PR positive	333 (46.8%)	203 (45.9%)	247 (60.2%)
Missing	12 (1.7%)	114 (25.8%)	7 (1.7%)
**Her2 status**			
Her2 Negative	561 (78.8%)	376 (85.1%)	217 (52.9%)
Her2 Positive	140 (19.7%)	43 (9.7%)	74 (18.0%)
Missing	11 (1.5%)	23 (5.2%)	19 (29.0%)

*ER status was determined by 10% positively stained nuclei from independent histological scoring.

Supplementary Table S2: Association of PRDX1 expression with clinicopathological parameters in the RPPA cohort (cohort 1)

Variables	PRDX1 Protein Expression#		*p*-value
	Low (n=356)	High (n=356)	
**Age at Diagnosis**			*<0.001*
≤50	93 (33.5)	61 (20.5)	
>50	185 (66.5)	237 (79.5)	
Missing	78	58	
**Tumor Size**			0.138
≤2cm	91 (38.7)	88 (46.3)	
>2cm	144 (61.3)	102 (53.7)	
Missing	121	166	
**Tumor Grade**			*<0.001**
I	25 (10)	40 (19.2)	
II	71 (28.5)	78 (37.5)	
III	153 (153)	90 (43.3)	
Missing	107	148	
**Nodal status**			0.07
N0	124 (48.4)	159 (56.4)	
N1+	132 (51.6)	123 (43.6)	
Missing	100	74	
**ER status**			*0.05*
ER negative	143 (41.7)	119 (34.2)	
ER positive	200 (58.3)	229 (65.8)	
Missing	13	8	
**PR status**			0.291
PR negative	190 (54.4)	177 (50.4)	
PR positive	159 (45.6)	174 (49.6)	
Missing	7	5	
**Her2 status**			*0.001*
FISH negative	262 (75.1)	299 (84.9)	
FISH positive	87 (24.9)	53 (15.1)	
Missing	7	4	

*Linear-by-linear χ^2 analysis; Others by Fisher's Exact test
#PRDX1 protein expression levels are stratified by the median protein expression value.

Supplementary Table S3: Association of PRDX1 expression with clinicopathological parameters in the consecutive cohort (cohort 2)

**Variables**	**PRDX1 Protein Expression#**		*p*-value
	**Low (n=221)**	**High (n=221)**	
**Age at Diagnosis**			0.066
≤50	42 (19.0)	27 (12.2)	
>50	179 (81.0)	194 (87.8)	
**Tumor Size**			*0.011*
≤2cm	120 (54.3)	147 (66.5)	
>2cm	101(45.7)	74 (33.5)	
**Histological type**			*0.003**
Indeterminate	13 (5.9)	14 (6.3)	
Ductal	159 (71.9)	131 (59.3)	
Lobular	23 (10.4)	45 (20.4)	
Tubular	10 (4.5)	18 (8.1)	
Medullary	11 (5.0)	3 (1.4)	
Mucinous	5 (2.3)	10 (4.5)	
**Tumor Grade**			*0.002**
I	40 (18.2)	67 (30.3)	
II	88 (40.0)	92 (41.6)	
III	92 (41.8)	62 (28.1)	
Missing	1	0	
**Nodal status**			0.755
N0	126 (63.3)	120 (61.5)	
N1+	73 (36.7)	75 (38.5)	
Missing	22	26	
**ER status**			*0.043*
ER negative	40 (19.2)	25 (11.9)	
ER positive	168 (80.8)	185 (88.1)	
Missing	13	11	
**PR status**			0.307
PR negative	68 (41)	57 (35.2)	
PR positive	98 (59)	105 (64.8)	
Missing	55	60	
**Her2 status**			0.151
0 - 2 +	183 (87.6)	193 (91.9)	
3+	26 (12.4)	17 (8.1)	
Missing	12	11	
**Ki67 status**			*0.004*
0 – 10%	60 (29.3)	90 (43.3)	
11 - 100%	145 (70.7)	118 (56.7)	
Missing	16	13	

*Linear-by-linear χ^2 analysis; Others by Fisher's Exact test
#PRDX1 protein expression levels are stratified by the median protein expression value.

Supplementary Table S4: Proteins that correlate with PRDX1 protein expression in the ER-positive and/or ER-negative cohort

	Protein	ER-positive (n = 304)		ER-negative (n = 99)	
		**p**	**r**	**p**	**r**
**Correlate in ER-positive tumors only**	ACC-pS79	*0.045*	0.115	0.063	-0.188
	ACC1	*0.000*	0.288	0.985	-0.002
	**Akt-pS473**	***0.011***	**-0.146**	**0.254**	**-0.116**
	**Akt-pT308**	***0.001***	**-0.190**	**0.805**	**0.025**
	B.Raf	*0.012*	0.143	0.455	-0.076
	Beclin	*0.011*	-0.146	0.322	0.101
	Bid	*0.000*	-0.233	0.295	-0.106
	Bim	*0.000*	0.199	0.055	0.193
	c.Met	*0.000*	-0.314	0.054	-0.194
	c.Met-pY1235	*0.002*	-0.177	0.138	0.150
	CDK1	*0.027*	-0.127	0.294	0.107
	Chk2	*0.000*	0.229	0.503	-0.068
	cIAP	*0.001*	0.192	0.267	0.113
	Collagen-VI	*0.000*	-0.232	0.136	-0.151
	COX.2	*0.002*	-0.174	0.355	0.094
	Cyclin-B1	*0.002*	0.177	0.312	0.103
	Cyclin-E1	*0.000*	0.264	0.087	0.173
	**E.Cadherin**	***0.007***	**0.154**	**0.255**	**-0.116**
	eEF2	*0.000*	0.237	0.264	-0.113
	EGFR	*0.000*	-0.240	0.410	-0.084
	EGFR-pY1068	*0.011*	-0.145	0.287	-0.108
	EGFR-pY1173	*0.000*	-0.218	0.921	-0.010
	EGFR-pY992	*0.001*	-0.198	0.566	-0.058
	**ER-alpha**	***0.024***	**0.129**	**0.707**	**0.038**
	FAK	*0.011*	-0.146	0.102	-0.165
	Fibronectin	*0.007*	-0.153	0.171	0.139
	FOXO3a	*0.023*	-0.130	0.648	-0.046
	GSK3.alpha.beta	*0.000*	0.325	0.329	-0.099
	HER3-pY1298	*0.009*	-0.150	0.241	-0.119
	HSP70	*0.000*	-0.265	0.942	-0.007
	IRS1	*0.000*	-0.247	0.090	-0.171
	K.Ras	*0.001*	-0.192	0.731	-0.035
	**MAPK-pT202-Y204**	***0.002***	**-0.179**	**0.096**	**-0.168**
	MIG.6	*0.001*	0.186	0.388	0.088
	Mre11	*0.002*	-0.174	0.120	0.157
	NF2	*0.038*	0.119	0.145	0.148
	Notch1	*0.018*	-0.136	0.761	-0.031
	p21	*0.025*	-0.129	0.111	-0.161
	p38-MAPK	*0.002*	0.177	0.235	0.120
	p70S6K	*0.039*	0.119	0.989	0.001
	PARP-cleaved	*0.027*	-0.127	0.152	0.145
	PDK1-pS241	*0.004*	0.163	0.928	-0.009
	PI3K.p110.alpha	*0.000*	0.334	0.638	0.048
	PKC.alpha	*0.041*	-0.117	0.281	-0.109
	PKC.alpha-pS657	*0.016*	-0.138	0.451	-0.077
	Rb	*0.000*	-0.222	0.671	0.043
	S6	*0.000*	0.248	0.215	0.126
	S6-pS240-S244	*0.028*	0.126	0.811	-0.024
	Src-pY527	*0.000*	-0.206	0.621	-0.050
	STAT3-pY705	*0.000*	-0.202	0.401	0.085
	Stathmin	*0.002*	-0.179	0.239	0.119
	Syk	*0.003*	0.172	0.115	0.159
	VEGFR2	*0.000*	0.271	0.774	0.029
	X4E.BP1	*0.000*	0.200	0.566	0.058
	XBP1	*0.000*	-0.204	0.297	-0.106
	YAP	*0.000*	-0.202	0.722	-0.036

**Correlate in opposite direction across both cohorts**	C.Raf	*0.004*	0.163	*0.011*	-0.254
	C.Raf-pS338	*0.005*	-0.162	*0.015*	0.244
	CD31	*0.043*	-0.116	*0.004*	0.288
	Chk1-pS345	*0.004*	-0.164	*0.011*	0.255
	mTOR	*0.000*	0.224	*0.039*	-0.208
	Rab25	*0.005*	-0.160	*0.003*	0.295
	SETD2	*0.047*	-0.114	*0.002*	0.306
	Snail	*0.005*	-0.161	*0.006*	0.274
**Correlate in the same direction across both cohorts**	A.Raf-pS299	*0.000*	0.272	*0.001*	0.320
	Bax	*0.003*	0.172	*0.001*	0.337
	Bcl.xL	*0.004*	0.165	*0.001*	0.334
	c.Jun-pS73	*0.000*	-0.315	*0.002*	-0.312
	c.Myc	*0.000*	-0.260	*0.001*	-0.315
	Caspase.7-cleavedD198	*0.018*	0.136	*0.000*	0.344
	Caveolin.1	*0.010*	-0.148	*0.021*	-0.232
	CD49b	*0.019*	0.135	*0.045*	0.202
	Claudin.7	*0.000*	0.284	*0.023*	0.228
	DJ.1	*0.002*	0.177	*0.003*	0.292
	eIF4E	*0.000*	0.214	*0.011*	0.255
	MEK1-pS217-S221	*0.034*	-0.121	*0.001*	-0.323
	Notch3	*0.000*	-0.218	*0.024*	-0.226
	Paxillin	*0.023*	-0.130	*0.000*	-0.500
	PCNA	*0.000*	0.276	*0.002*	0.305
	PTCH	*0.000*	-0.210	*0.001*	-0.343
	RBM3	*0.000*	0.215	*0.026*	0.224
	Smac	*0.000*	0.351	*0.003*	0.298
	Transglutaminase	*0.018*	0.135	*0.008*	0.266
	VASP	*0.000*	0.265	*0.000*	0.389
	XIAP	*0.001*	-0.190	*0.022*	-0.231
	XRCC1	*0.001*	0.197	*0.008*	0.265
**Correlate in ER-negative tumors only**	X53BP1	0.695	-0.023	*0.005*	-0.282
	Akt	0.790	0.015	*0.002*	-0.311
	AMPK-pT172	0.671	0.024	*0.050*	-0.198
	ATM	0.749	0.018	*0.050*	-0.198
	beta.Catenin	0.363	0.052	*0.006*	-0.274
	Caspase.8	0.391	0.049	*0.048*	0.199
	Chk1	0.142	-0.084	*0.001*	0.324
	eEF2K	0.817	-0.013	*0.015*	-0.243
	ERK2	0.516	0.037	*0.000*	-0.350
	GAB2	0.067	0.105	*0.014*	-0.245
	GSK3.alpha.beta-pS21-S9	0.634	-0.027	*0.005*	-0.279
	IGF.1R.beta	0.856	0.010	*0.011*	-0.255
	Ku80	0.405	0.048	*0.000*	-0.362
	LBK1	0.203	-0.073	*0.045*	0.202
	Lck	0.405	0.048	*0.020*	0.234
	MSH2	0.208	0.072	*0.019*	-0.236
	p27-pT157	0.284	-0.062	*0.044*	0.203
	p90RSK-pT359-S363	0.101	-0.094	*0.001*	-0.317
	Pea.15	0.893	0.008	*0.000*	0.345
	Rad50	0.832	-0.012	*0.030*	-0.218
	Rad51	0.685	-0.023	*0.010*	0.256
	Src-pY416	0.404	-0.048	*0.001*	0.325
	STAT5.alpha	0.379	-0.051	*0.002*	-0.310
	Tuberin	0.703	0.022	*0.000*	-0.444
	X14.3.3-epsilon	0.143	0.084	*0.002*	0.313
	X4E.BP1-pT37	0.353	-0.053	*0.013*	-0.248

Significant value ($p<0.05$) highlighted in *italics*. Pearson's correlation values represented by r.
