# Supplementary figures and images for: Peroxiredoxin-1 protects estrogen receptor α from oxidative stress-induced suppression and is a protein biomarker of favorable prognosis in breast cancer
Source: Breast Cancer Res. 2014 Jul 10;16(4):R79. doi: 10.1186/bcr3691 (PMC4226972; doi:10.1186/bcr3691)

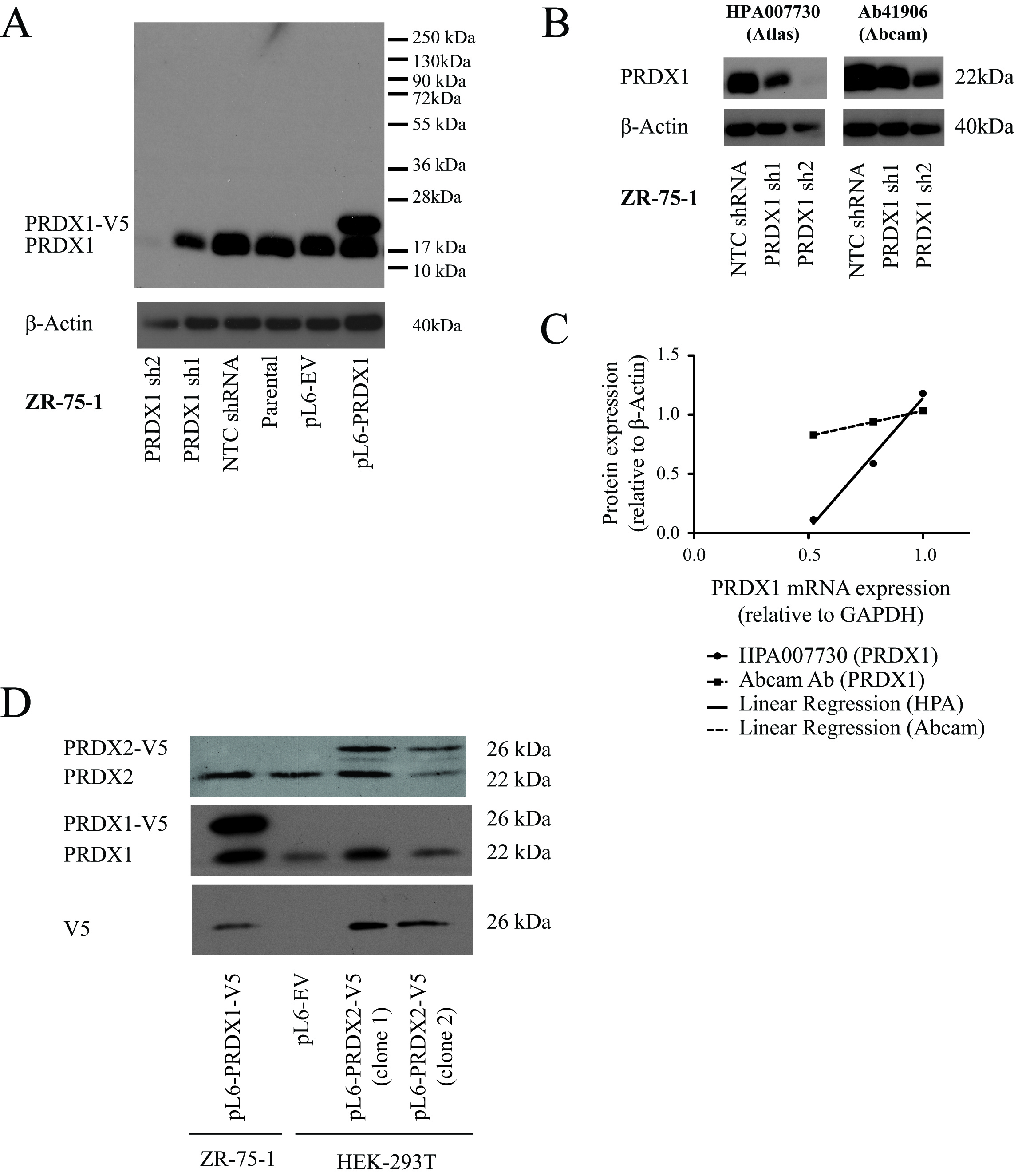

Supplement: Additional file 3: Figure S1 — Additional antibody validation experiments. (A) Full length immunoblotting gel shows a single discrete signal, the intensity of which correlates with PRDX1 knockdown/overexpression across recombinant ZR-75-1 cell lines (V5-tagged PRDX1 protein runs at 25 kDa, with native protein at 22 kDa). (B) Immunoblotting signal in PRDX1-silenced ZR-75-1 cell lines using independent PRDX1-targeting antibodies. (C) Protein and transcript expression were compared across three recombinant ZR-75-1 cells lines (NTC shRNA, PRDX1-sh1, PRDX1-sh2), which shows a greater correlation using the antibody from Atlas antibodies (solid line) compared to the Abcam antibody (dotted line). (D) Overexpression of PRDX1 in ZR-75-1 cells did not affect PRDX2 protein expression, while PRDX2 overexpression in T47D cells did not affect PRDX1 protein expression. NTC, non-targeting control; PRDX1, peroxiredoxin 1; shRNA, short hairpin loop RNA. [file bcr3691-S3.jpeg]

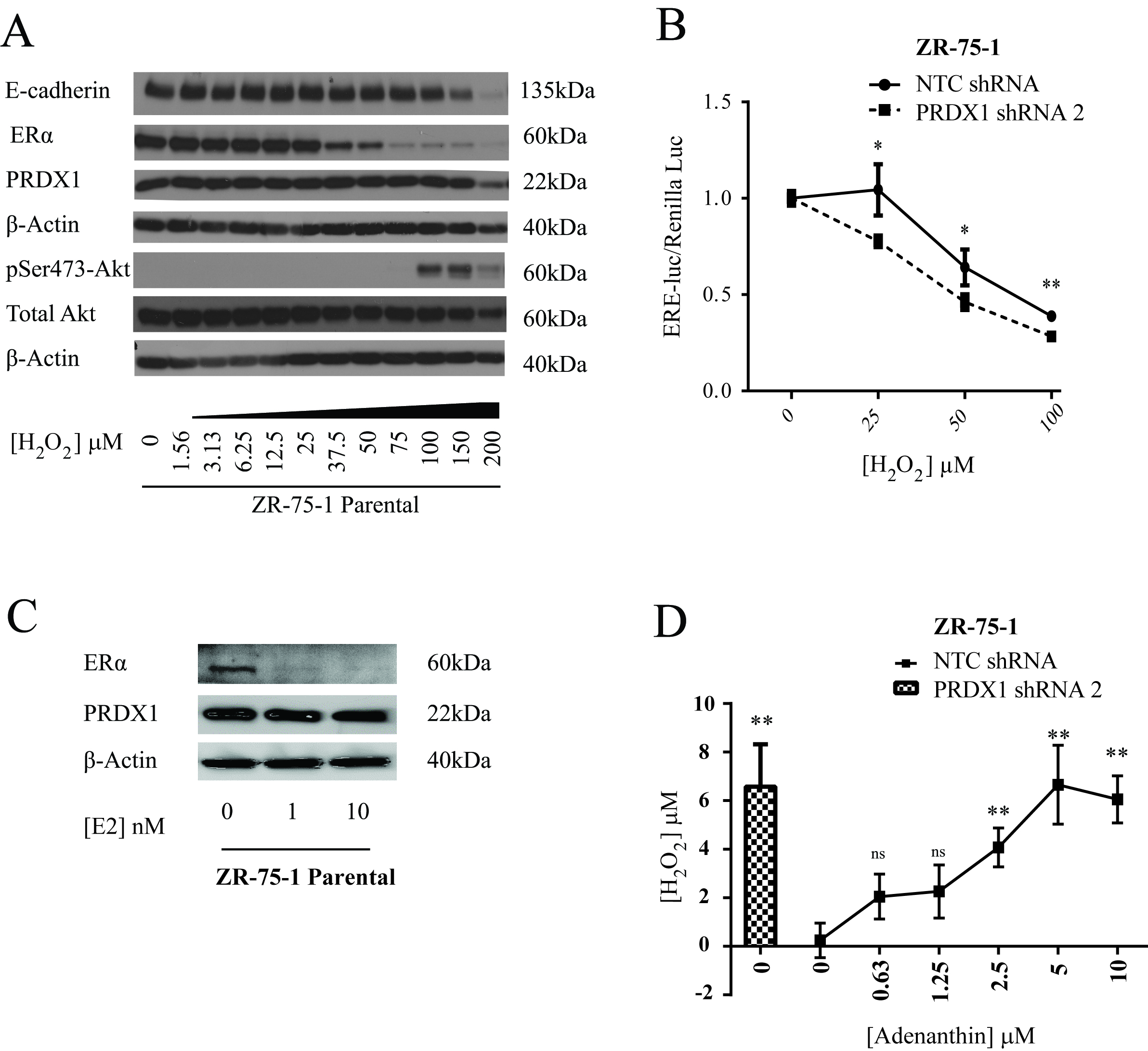

Supplement: Additional file 4: Figure S2 — Additional in vitro Western blotting and functional assays. (A) Effect of H2O2 on ERα, pSer473-Akt and E-cadherin levels, along with respective loading controls. Parental ZR-75-1 cells were treated with increasing levels of H2O2 for 16 hours. (B) PRDX1 knockdown enhances the H2O2-mediated suppression of ER activity. Relative units of the ERE-luciferase reporter expression were normalized to Renilla-luciferase units. H2O2 treatment was 16 hours in duration. (C) PRDX1 protein expression is not driven by ER activity. Western blot analysis shows that no change in PRDX1 protein expression following stimulation of parental ZR-75-1 cells with 1 or 10 nM 17-β estradiol for 48 hours. During this experiment, these cells were cultured in DMEM containing 0.1% FBS supplemented with 17-β estradiol or vehicle control. (D) Treatment with adenanthin inhibits metabolism of H2O2 following three hours treatment with 50 μM H2O2. Knockdown of PRDX1 (sh2) is shown as a positive control. (ns: non-significant; *P <0.05; **P <0.01). DMEM, Dulbecco’s modified Eagle’s medium; ER, estrogen receptor; ERE, estrogen transcriptional response element; FBS, fetal bovine serum; H2O2, hydrogen peroxide; PRDX1, peroxiredoxin 1. [file bcr3691-S4.jpeg]

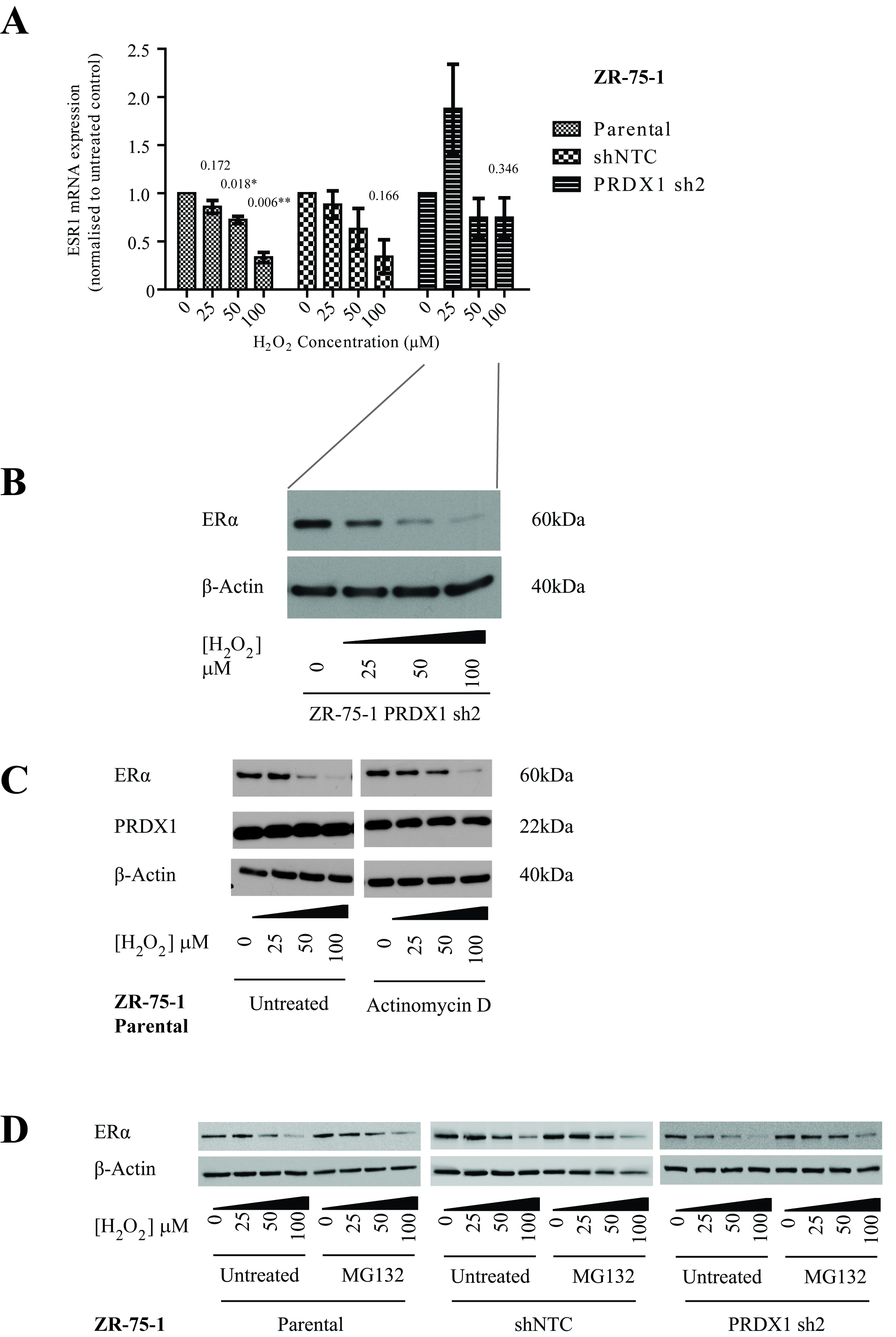

Supplement: Additional file 5: Figure S3 — Effects on mRNA expression and protein stability after H2O2 treatment. (A)ESR1 mRNA expression is suppressed after induction of oxidative stress (16 hours H2O2 treatment) in ZR-75-1 cells. Silencing of PRDX1 does not enhance this oxidative stress-mediated suppression of ESR1 mRNA. Error bars represent the SEM from independent biological experiments (*P <0.05; **P <0.01). (B) H2O2 treatment suppresses ERα protein expression in these PRDX1-silenced ZR-75-1 cells. (C) Inhibition of transcription (1 μg/μl Actinomycin D) does not alter H2O2-mediated suppression of ERα protein. (D) Inhibition of proteasomal degradation (5 μM MG132) prevents oxidative stress-induced ERα suppression in ZR-75-1 cells. After inhibition of proteasomal degradation, PRDX1-sh2 expressing cells display a weaker H2O2-mediated suppression. All treatments were 16 hours in duration. ER, estrogen receptor; H2O2, hydrogen peroxide; PRDX1, peroxiredoxin 1. [file bcr3691-S5.jpeg]

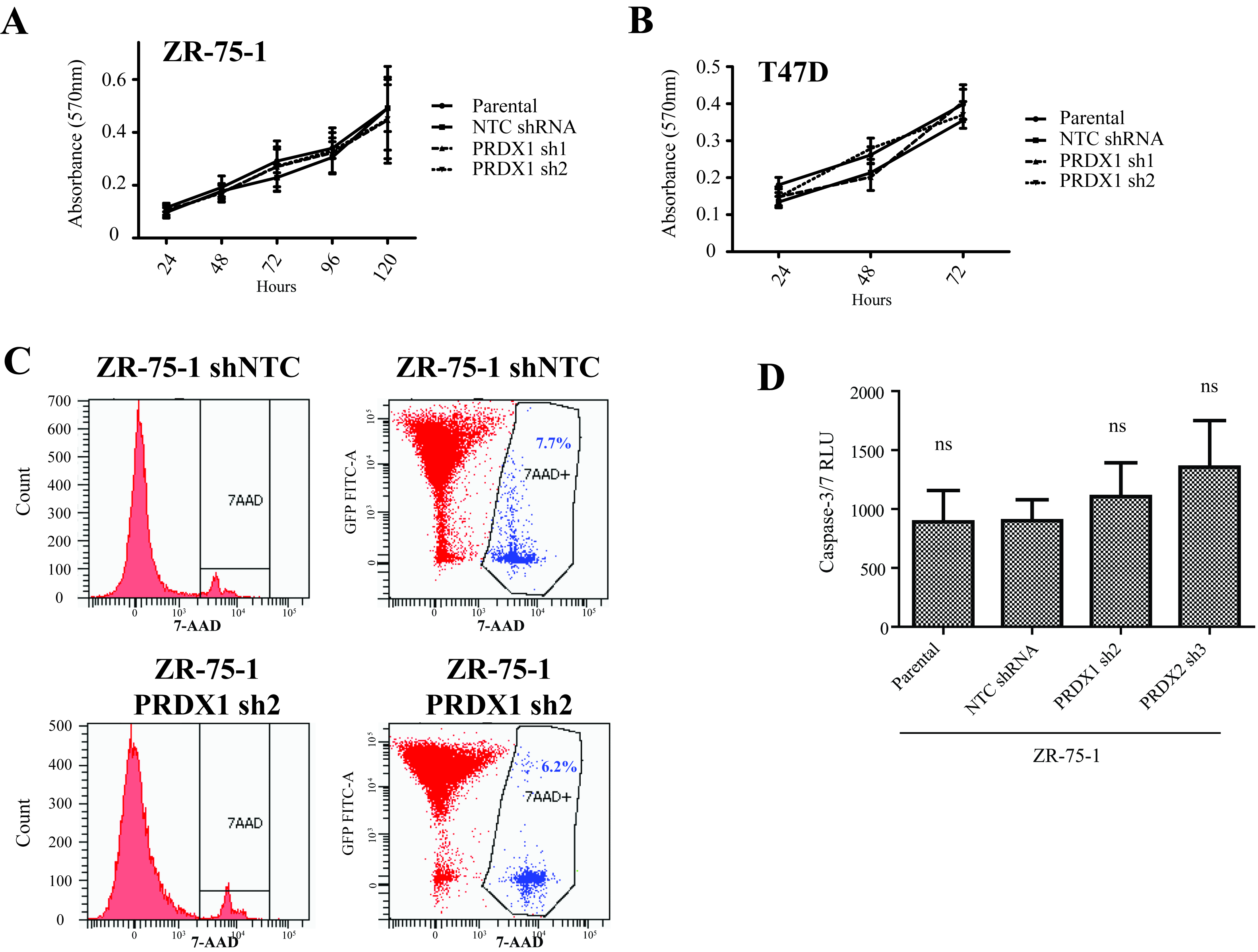

Supplement: Additional file 6: Figure S4 — Viability and apoptosis assays. PRDX1 knockdown does not alter cell growth or viability in ZR-75-1 and T47D breast cancer cell lines. ZR-75-1 (A) and T47D (B) cells were grown in normal medium for up to 120 hours, with cell viability (MTT assay) being measured every 24 hours. Experiments were repeated three times with six wells per experiment. Error bars represent the SEM. (C) Flow cytometry viability analysis (7-AAD viability staining solution) was also used to demonstrate this lack of change in proliferation. (D) The Apotox-Glo™ Triplex assay kit was used to measure caspase 3/7 activity in PRDX1 or PRDX2-silenced ZR-75-1 cells compared to the parental and NTC shRNA controls. Error bars represent the SD (ns: non-significant). PRDX1, peroxiredoxin 1. [file bcr3691-S6.jpeg]

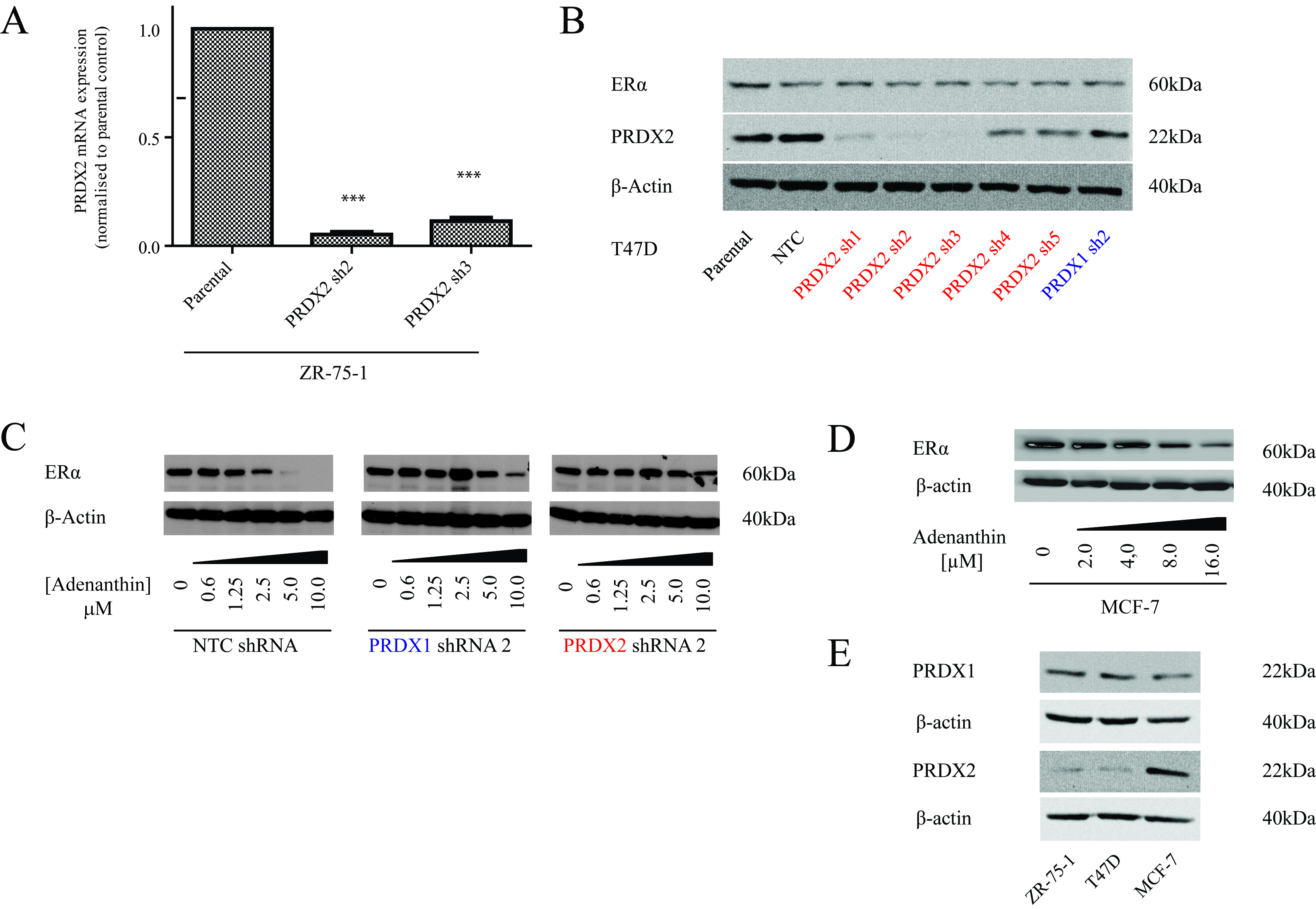

Supplement: Additional file 7: Figure S5 — PRDX2 RT-PCR and Western blotting, including adenanthin treatment of PRDX2-silenced cells. (A) PRDX2 mRNA and (B) protein expression is reduced in ZR-75-1 and T47D cells after lentiviral transduction of up to five different anti-PRDX2 shRNAs. (C) Silencing of PRDX1 or PRDX2 abrogates the adenanthin-induced suppression of ERα protein in ZR-75-1. All treatments were 16 hours in duration. Error bars represent the SEM from two independent experiments (***P <0.001). ER, estrogen receptor; PRDX1, peroxiredoxin 1; RT-PCR, real-time polymerase chain reaction; shRNA, short hairpin loop RNA. [file bcr3691-S7.jpeg]
